# Transcriptomic Analysis of Mature Transgenic Poplar Expressing the Transcription Factor *JERF36* Gene in Two Different Environments

**DOI:** 10.3389/fbioe.2022.929681

**Published:** 2022-06-14

**Authors:** Weixi Zhang, Yanbo Wang, Tengqian Zhang, Jing Zhang, Le Shen, Bingyu Zhang, Changjun Ding, Xiaohua Su

**Affiliations:** ^1^ State Key Laboratory of Tree Genetics and Breeding, Research Institute of Forestry, Chinese Academy of Forestry, Beijing, China; ^2^ Key Laboratory of Tree Breeding and Cultivation, National Forestry and Grassland Administration, Beijing, China; ^3^ Nanchang Institute of Technology, Nanchang, China; ^4^ Co-Innovation Center for Sustainable Forestry in Southern China, Nanjing Forestry University, Nanjing, China

**Keywords:** transgenic poplar, stress resistance, transcriptome analysis, differentially expressed genes, environmental effect

## Abstract

During the last several decades, a number of transgenic or genetically modified tree varieties with enhanced characteristics and new traits have been produced. These trees have become associated with generally unsubstantiated concerns over health and environmental safety. We conducted transcriptome sequencing of transgenic *Populus alba* × *P. berolinensis* expressing the transcription factor JERF36 gene (ABJ01) and the non-transgenic progenitor line (9#) to compare the transcriptional changes in the apical buds. We found that 0.77% and 1.31% of the total expressed genes were significant differentially expressed in ABJ01 at the Daqing and Qiqihar sites, respectively. Among them, 30%–50% of the DEGs contained cis-elements recognized by *JERF36*. Approximately 5% of the total number of expressed genes showed significant differential expression between Daqing and Qiqihar in both ABJ01 and 9#. 10 DEGs resulting from foreign gene introduction, 394 DEGs that resulted solely from the environmental differences, and 47 DEGs that resulted from the combination of foreign gene introduction and the environment were identified. The number of DEGs resulting from environmental factors was significantly greater than that resulting from foreign gene introduction, and the combined effect of the environmental effects with foreign gene introduction was significantly greater than resulting from the introduction of *JERF36* alone. GO and KEGG annotation showed that the DEGs mainly participate in the photosynthesis, oxidative phosphorylation, plant hormone signaling, ribosome, endocytosis, and plant-pathogen interaction pathways, which play important roles in the responses to biotic and abiotic stresses ins plant. To enhance its adaptability to salt-alkali stress, the transgenic poplar line may regulate the expression of genes that participate in the photosynthesis, oxidative phosphorylation, MAPK, and plant hormone signaling pathways. The crosstalk between biotic and abiotic stress responses by plant hormones may improve the ability of both transgenic and non-transgenic poplars to defend against pathogens. The results of our study provide a basis for further studies on the molecular mechanisms behind improved stress resistance and the unexpected effects of transgenic gene expression in poplars, which will be significant for improving the biosafety evaluation of transgenic trees and accelerating the breeding of new varieties of forest trees resistant to environmental stresses.

## Introduction

During the last several decades, the revolutionary scientific advances in the fields of plant genetics and molecular biology has made it possible to genetically regulate tree metabolic pathways in a highly targeted manner, resulting in new varieties, some of which could not be produced by traditional breeding. As a result, a large number of transgenic or genetically modified (GM) tree varieties with enhanced characteristics and new traits have been produced. Examples are *Eucalyptus* (Harcourt et al., 2000; Matsunaga et al., 2012), pine (*Pinus radiata*), cork oak (*Quercus suber*), poplar (*Populus* spp.), blueberry (*Vaccinium corymbosum*), strawberry (*Fragaria* × *ananassa*), apple (*Malus* × *domestica*), and papaya (*Carica papaya*) (Grace et al., 2005; Alvarez et al., 2009; Li et al., 2009; Klocko et al., 2018; Hikosaka et al., 2019; Zong et al., 2019). Some of these have been commercialized around the world. Due to its mature regeneration tissue culture system and smaller genome, poplar has become a model species for the genetic engineering of trees. The improved traits of transgenic trees have mainly focused on insect resistance, disease resistance, herbicide resistance, salt tolerance, drought tolerance, material improvement, and development regulation.

Even though transgenic trees were originally developed for purposes of global economic benefit, they later become associated with generally unsubstantiated concerns over health and environmental safety, which were then transformed into political issues. For this reason, many studies have examined the safety of transgenic plants with respect to food, feed, and the environment, and have also monitored changes in the genome, transcriptome, proteome, and metabolome of transgenic crop plants in molecular breeding programs (Filipecki and Malepszy. 2006; Mao et al., 2016; Zhou et al., 2016). A large number of studies have shown that in genetically modified organisms, when a foreign gene is inserted into the genome, the original genetic information of the host is disrupted, and the expression of endogenous genes may be changed due to transformation effects, positional effects, recombination effects, insertion effect, and induced effects (Filipecki and Malepszy, 2006; Metzdoff et al., 2006). The inadvertent changes in transgenic plants caused by transformation effects or the tissue culture process may further lead to changes in the metabolic pathways, which in turn lead to unintended changes in intrinsic plant traits and properties that have been observed in transgenic plants (Ladics et al., 2015; Schnell et al., 2015). Such unintended changes are not easily anticipated and are difficult to detect, raising caution when assessing the risks associated with GM plants (Ladics et al., 2015; Schnell et al., 2015; Wang et al., 2018).

With the rapid development of high-throughput DNA sequencing technology, advancements in “omics” technologies such as transcriptomics have been shown to be powerful new techniques for identifying gene expression changes at the whole genome level, including the effects of introduced genes and environmental interactions on gene expression, as well as the unintended effects in GM plants (Kuiper et al., 2001; Ouakfaoui and Miki, 2005; Ricroch et al., 2011; Gong and Wang, 2013; Herman and Price, 2013; Pauwels et al., 2015; Wang et al., 2018; Tan et al., 2019; Liu et al., 2020). “Omics” technologies such as genomics, transcriptomics, and metabolomics have been applied to transgenic rice, maize, soybean, barley, potato and pigeon pea to detect and identify the differences between transgenic and non-transgenic plants at the whole genome scale, as well as to analyze the causes of these differences. (Kuiper et al., 2001; Ouakfaoui and Miki, 2005; Ricroch et al., 2011; Gong and Wang, 2013; Herman and Price, 2013; Wang et al., 2018; Debode et al., 2019; Tan et al., 2019). These studies have revealed certain differences in transcriptomics and metabolomics of the tested crops, and Fonseca et al. (2015) show that the major factor influencing transgenic vs. non-transgenic plants may be the *in vitro* culture stress imposed during plant transformation and selection. Another study demonstrated that environmental stress may also be a major cause of proteomic/transcriptomic changes rather than transgenesis, and the differences that occur during genetic modification are mainly short-term physiological changes that are attenuated in subsequent generations (Batista et al., 2017). Liu et al. (2020) and Wang et al. (2018) have suggested that the changes brought about by transgenesis were less than those due to natural variation among varieties of maize and rice.

However, compared with crops, there are relatively few studies on genetically modified trees. Fang et al. (2016) compared the transcriptome of transgenic papaya to its non-transgenic progenitor, and 842 differentially expressed genes (DEGs) out of 20,700 transcripts were identified between the two cultivars. The upregulated DEGs in transgenic papaya, which were mainly related to various transcription factors, transporters, and hormone biosynthesis, may increase PRSV resistance in the GE papaya plants. Our laboratory used transcriptomic and metabolomic analyses to compare the differences between muti-gene transgenic poplar (*P.* × *euramericana* ‘Guariento’) and its non-transgenic progenitor in seedlings and adult plants (Zhang, et al., 2014; Ning et al., 2018). Our results showed that while 782 DEGs were identified between the transgenic and non-transgenic plants, only 197 genes were associated with plant stress tolerance (biological and abiotic) functions. In addition, we also identified 197 metabolites that showed differential abundances between the genetically modified and non-transgenic poplar plants. We also found that the levels of some metabolites involved in growth, stress-related processes, and insect resistance differed greatly between the transgenic and non-transgenic poplars. Therefore, we assume that the significant differences expression of other functional genes or metabolites involved in other Metabolic may be associated with environmental factors. and the remaining differently expression genes need to be further studied.

Hybrid *P. alba* × *P. berolinensis* plants exhibit fast growth and a beautiful tree shape, and are widely used in urban greening and afforestation in the northeast, northwest, and north of China. Base on its susceptibility to salt stress, *P. alba* × *P. berolinensis* is difficult to grow in saline soils, which seriously affects its cultivation on saline-alkali land (Ding et al., 2020). In the early stage of our research, we created an improved, salt-tolerant transgenic line of *P. alba* × *P. berolinensis* by overexpressing *JERF*36, a gene from tomato that encodes a jasmonate/ethylene-responsive transcription factor (Li et al., 2009; Zhang and Huang, 2010). A previous study showed that, compared with the non-transgenic poplar trees (9#), 4-year-old transgenic *P. alba* × *P*. *berolinensis* trees (ABJ01) grew much faster and showed significantly enhanced salt tolerance, and that this was due to improved maintenance of the K^+^/Na^+^ balance in the cytoplasm. However, a genome-wide gene expression study of transgenic *Populus alba* × *P*. *berolinensis* trees has not yet been performed, and because these are long-lived perennial plants, they should be tested over many years.

In the present study, transcriptomics was used to analyze the differential expression of genes in adult transgenic poplar plants in different environments, and to examine the gene expression patterns and quantify the unintended effects in transgenic poplar. Our results establish a foundation for the further improvement of safety evaluation systems for transgenic trees and will help to accelerating the breeding of new varieties of forest trees resistant to environmental stresses.

## Materials and Methods

### Plant Material

The transcriptomes of mature plants of the transgenic (ABJ01) and the non-transgenic progenitor clone (9#) of hybrid poplar (*P. alba* × *P*. *berolinensis*) were compared in this study. The transgenic line expresses *JERF36*, a tomato gene that encodes a jasmonate/ethylene-responsive AP2/EREBP family transcription factor, that is, related to plant stress resistance. The neomycin phosphotransferase II gene (*NPT* II) derived from the *E. coli* transposon Tn5 was used as a marker for kanamycin resistance (Li et al., 2009). Trees were planted in two experimental fields in different environments established in Heilongjiang province in northeast China in 2007 and 2009; a saline site, in Daqing (46^o^34′N, 125^o^08′E; D), with salt content of soil is about 0.2%–0.4% and a non-saline site in Qiqihar (47^o^27′N, 122^o^51′E; Q). Both test sites are located in the Songnen Plain, which has a temperate continental monsoon climate, a mean annual temperature of 4°C, annual precipitation of 415 mm, and an elevation of 146 m above sea level. The trees in the experimental forests were mature and of similar ages (8–9 years old). The diameter at breast height (DBH) and survival rate of ABJ01 and 9# in Daqing saline site was about 16.12 cm, 50% (ABJ01) and 15.57 cm, 25% (9#), respectively. And the DBH of which was 15.33 cm and 15.02 cm in Qiqihar with the survival rate more than 90%. In March 2016, three blocks were selected as three biological replicates from two sites, and apical buds were collected randomly from the branches in four directions from the top 1/3 of the tree, three transgenic trees [ABJ01 (A)] and three non-transgenic trees [9# (B)] in each block were choose and combined into one biological replicate. In total, 12 samples were collected (3 × 4 bud samples: DA, DB, QA, QB). All samples were flash frozen in liquid nitrogen, and were then transferred to a −80°C freezer until required for RNA extraction.

### cDNA-Library Preparation and RNA Sequencing for Transcriptome Analysis

Total RNA was extracted from apical buds of transgenic (ABJ01) and non-transgenic poplar (9#) using a Plant RNA Extraction Kit (Autolab, China) following the manufacturer’s protocol. The concentration and quality of each RNA sample were determined using a NanoDrop 2000™ micro-volume spectrophotometer (Thermo Scientific, Waltham, MA, United States) and an Agilent 2100 Bioanalyzer (Agilent Technologies Inc., Palo Alto, CA, United States). Two methods were used to treat the total RNA: 1) oligo (dT) magnetic beads were used to select mRNA with polyA tails, and 2) the rRNA was hybridized with a DNA probe and the DNA/RNA hybrids were digested with DNase I to remove the DNA probe. The purified mRNA was then fragmented, and double-stranded cDNA (ds cDNA) was synthesized using reverse transcriptase and 6-mer random primers. The ds cDNA was phosphorylated at the 5′ end and a single dA was added to the 3′ end, and the ds cDNA fragments were then ligated to adaptors with dT at the 3′ ends. Two specific primers were used to amplify the ligation products. The PCR products were heat denatured and the single-stranded DNA was cyclized by splint oligo and DNA ligase, and then sequenced on the BGISEQ-500 platform. Prior to downstream processing, the raw reads were initially processed to obtain clean reads by removing adaptor sequences, reads in which the number of unknown bases was >10%, and low quality sequences in which >50% of the bases had quality scores ≤5.

### Sequencing Read Mapping and Quantification of Gene Expression

Bowtie2 software (Langmead et al., 2009) was used to map the clean reads to reference genes and the reads were mapped to the *P. trichocarpa* v3.0 reference genome (https://phytozome.jgi.doe.gov/pz/portal.html#!info?alias=Org_Ptrichocarpa) with HISAT (Kim et al., 2015). The reads from each biological replicate in all libraries were mapped independently, and reads that mapped to reference sequences from unigenes were used for further analysis.

RSEM (RNASeq by Expectation Maximization) was used to compute the maximum likelihood abundance estimates for accurate transcript quantification (Li and Dewey, 2011). Fragments per kilobase of exon per million fragments mapped (FPKM) were used to calculate expression levels. The FPKM method is able to eliminate the influence of different gene lengths and sequencing discrepancies on the calculation of relative gene expression. Therefore, the calculated gene expression levels can be directly used for comparing the differences in gene expression between samples. To avoid false positive estimations of gene expression, transcripts with FPKM values <1 in both libraries were not subjected to further analysis.

### Gene Ontology and Kyoto Encyclopedia of Genes and Genomes Orthology Enrichment Analyses of the Differentially Expressed Genes

Gene Ontology (GO) enrichment analysis was used to categorize the main biological functions of the DEGs. All DEGs were mapped to GO terms in GO database (http://www.geneontology.org/), gene numbers were calculated for every term, and a hypergeometric test was used to find significantly enriched GO terms in the input list of DEGs, based on GO: Term Finder. The calculated *p*-values were adjusted using the conservative Bonferroni Correction (Abdi, 2007), taking the corrected *p*-value ≤ 0.05 as a threshold. GO terms fulfilling this condition were defined as significantly enriched in the DEGs.

KEGG (Kyoto Encyclopedia of Genes and Genomes) was used to perform pathway enrichment analysis of the DEGs (Kanehisa et al., 2008). This analysis identifies significantly enriched metabolic pathways or signal transduction pathways in the DEGs compared with the whole genome background. The calculated *p*-values were adjusted using the Bonferroni Correction, taking the corrected *p*-value ≤ 0.05 as a threshold.

### Quantitative Real-Time

Total RNA was extracted from poplar buds using the RNeasy Plant Mini Kit (Qiagen, United States) according to the manufacturer’s instructions. The yield of RNA was determined using a NanoDrop 2000 spectrophotometer (Thermo Scientific, United States), and the integrity was evaluated using agarose gel electrophoresis and ethidium bromide staining. cDNA was synthesized using a PrimerScript RT reagent kit (TaKaRa, Dalian, China). Amplifications were performed using a LightCycler^®^ 480 II Real-time PCR Instrument (Roche, Swiss) with SYBR Green Realtime PCR Master Mix (Takara, Dalian, China).

The primer sequences were designed in the laboratory and synthesized by Generay Biotech (Generay, PRC) based on mRNA sequences obtained from the NCBI database (Supplementary Table S1). The expression levels of mRNAs were normalized to the expression of *UBQ-like* (GenBank Accession BU871588) and were calculated using the 2^−ΔΔCt^ method (Livak and Schmittgen, 2001).

## Results

### BGISEQ-500 RNA Sequencing and Alignment to the Reference Genome

**FIGURE 1:**
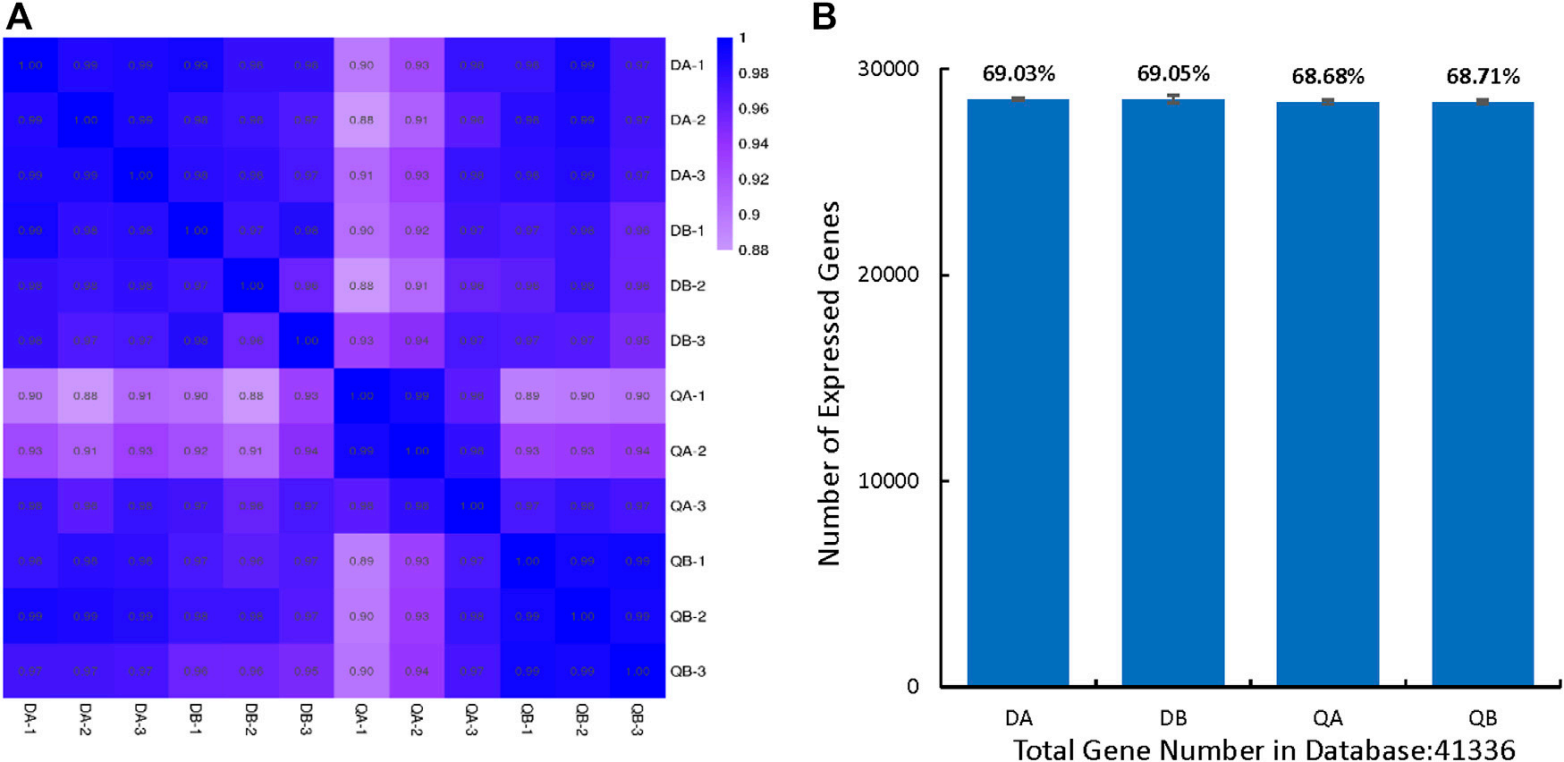
Overview of transgenic and non-transgenic poplars’ transcriptomes. **(A)** Pairwise correlation of different biological replicates from of transgenic (ABJ01) and non-transgenic (9#) using FPKM values. The color intensities (scale in the right side bar) and the numbers indicate the degree of pairwise correlation. **(B)** Number of expressed genes in Database of each line, The proportion at the top of each bar equals expressed genes number divided by total gene number reported in database. DA: ABJ01 from Daqing, DB: 9# from Daqing; QA: ABJ01 from Qiqihar, QB: 9 # from Qiqihar.

To characterize transcriptomic changes induced by *Agrobacterium* transformation and the different growing environments at the genome scale, 12 cDNA libraries were constructed from buds of ABJ01 (transgenic, A) and 9# (non-transgenic, B) poplar trees sampled from three blocks in two different environments and sequenced using BGISEQ-500 sequencing platform. RNA sequencing (RNA-seq) generated 289.65 million reads for a total of 14.48 Gb of raw data. More than 24.14 million reads were generated from each sample. After stringent quality assessment and data filtering, 289.55 million (99.96%) of the clean reads were selected for further analysis. Clean reads were mapped to the *Populus trichocarpa* reference genome v3.0. An average of 72.35% of the reads mapped to the genome, and an average of 40.88% mapped to unique positions (Supplementary Table S2). Only uniquely matched reads were used in the analysis of gene expression in the different cultivars. We compared the RNA-seq expression profiles to evaluate the correlation coefficients between pairwise comparisons of the three biological replicates (Figure 1A). This results indicated that estimated levels were highly consistent between any pair of replicates from each line (*r* = 0.91–0.99), and compared to the reference genes, >28,000 (68.87%) genes were identified in each line from the database (41,335 genes) (Figure 1B). These data indicate that the transcriptome data from the two samples and three replicates was sufficient for further analysis.

**FIGURE 2:**
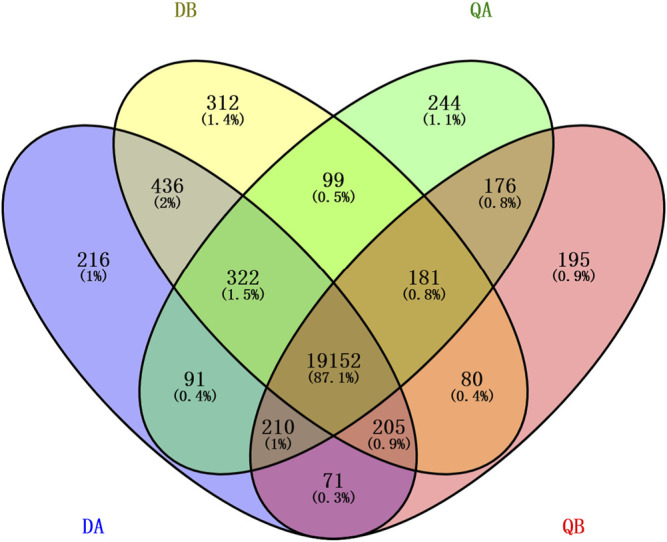
Venn diagram showing the differentially expressed genes (DEGs) in the transgenic (ABJ01) and non-transgenic (9#) poplars grown in Daqing and Qiqihar. DA: ABJ01 from Daqing, DB: 9# from Daqing; QA: ABJ01 from Qiqihar, QB: 9# from Qiqihar.

In this study, we considered the gene to be expressed when the FPKM value ≥1. There were 20,270–20,787 genes expressed in the transgenic (A) and non-transgenic (B) poplars grown in Daqing (D) and Qiqihar (Q), respectively. Among them, 19,719 genes were co-expressed in QA and QB, 20,125 genes were co-expressed in DA and DB, 19,775 genes were co-expressed in QA and DA, and 19,618 genes were co-expressed in QB and DB. In addition, we found that 19,152 (87.1%) genes were co-expressed in the four groups (DA, DB, QA, and QB), and only around 1% of the genes were expressed independently in each of the four groups (Figure 2).

### Analysis of Differentially Expressed Genes in the Transgenic and Non-Transgenic Poplar Trees Grown in the Different Environments

**FIGURE 3:**
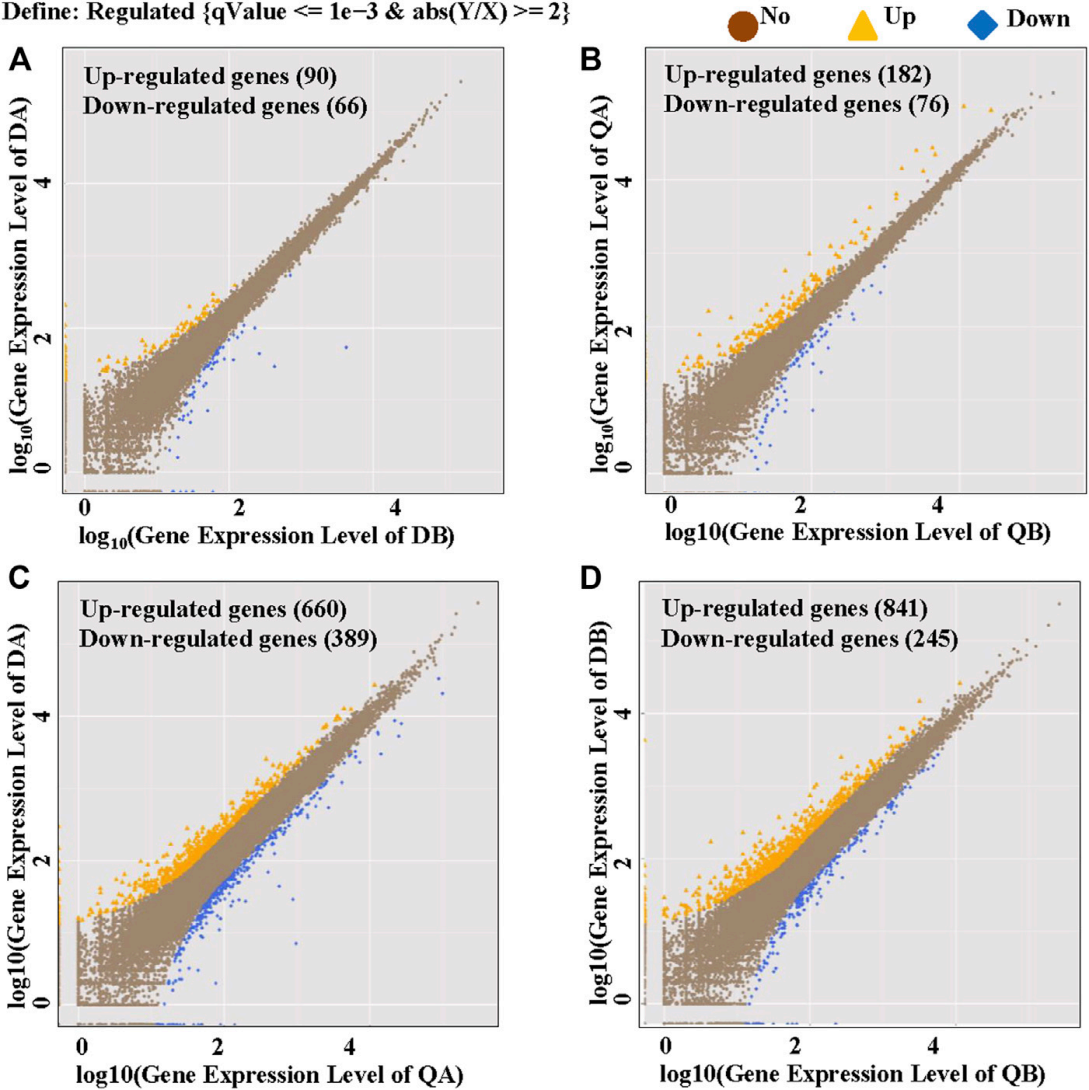
Identification of DGEs between the two poplar lines grown in the two different environments. Upregulated genes are shown in yellow, downregulated genes are shown in blue, and genes that are not differentially expressed are shown in brown. **(A,B)** The log_10_ (Gene Expression Level of ABJ01) (y-axis) plotted against the log_10_ (Gene Expression Level of 9#) (x-axis) in Daqing **(A)** and Qiqihar **(B)**. **(C,D)** The log_10_ (Gene Expression Level of ABJ01 or 9# from Daqing) (Y-axis) plotted against the log_10_ (Gene Expression Level of ABJ01 or 9# from Qiqihar) (x-axis); DA vs. QA **(C)** and DB vs. QB **(D)**.

To identify global transcriptional changes that occurred after insertion of the transgene, the gene expression profiles of the DEGs are expected to meet the following three criteria: 1) the FPKM value is ≥1 in either of the libraries, 2) the log_2_ fold-change of the expression ratio is ≥1 or ≤−1, and 3) the adjusted *p*-value is ≤ 0.05. To determine how many genes are significantly affected, a scatter plot was constructed by plotting Log_10_ Gene Expression Level of the four pairwise comparisons DA vs. DB, QA vs. QB, DA vs. QA, and DB vs. QB. As shown in Figure 3, the expression of most genes in the two lines (A and B) from the two fields was similar. Comparisons between ABJ01 (A) and 9# (B) from Daqing and Qiqihar, respectively, showed that 156 DEGs out of 20,125 co-expressed genes (0.77%) were identified in Daqing, of which 90 genes were upregulated and 66 were downregulated (Figure 3A). In addition, 258 DEGs out of 19,719 co-expressed genes (1.31%) were identified in Qiqihar, of which 182 genes were upregulated and 76 were downregulated (Figure 3B). Comparisons of ABJ01 or 9# between Daqing and Qiqihar had the largest number of DEGs. In total, there were 1,049 DEGs out of 19,775 co-expressed genes (5.30%) in ABJ01, of which 660 genes were upregulated and 389 were downregulated (Figure 3C), and 1,086 DEGs out of 19,618 co-expressed genes (5.54%) in 9#, of which 841 genes were upregulated and 245 were downregulated (Figure 3D). All of this data indicates that the numbers of DEGs between the transgenic and non-transgenic poplars from the two sites were far less than those between the different environments of ABJ01 and 9#, and the DEGs were mainly upregulated in the four comparison groups, particularly between the two different environments. Preliminary analysis suggests that environmental factors had more influence on gene expression than did the introduction of a foreign gene.

**FIGURE 4:**
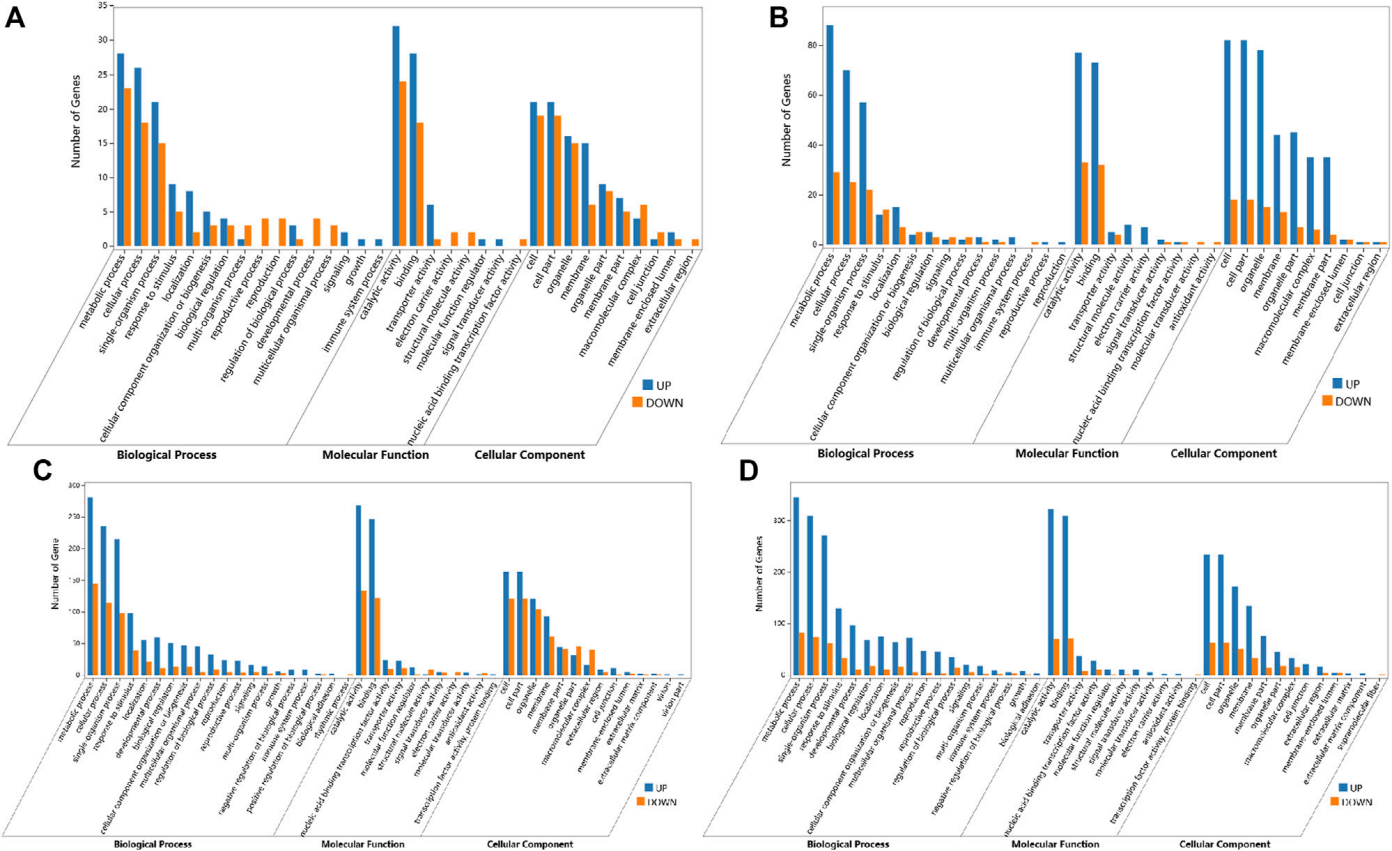
GO annotations of DEGs from comparisons of the ABJ01 and 9# transcriptomes for poplars grown in Daqing and Qiqihar. Level2 GO annotation of DEGs from the DA vs. DB comparison **(A)**; the QA vs. QB comparison **(B)**; the DA vs. QA comparison **(C)** and the DB vs. QB comparison **(D)**. The top GO terms in each comparison are shown for the three major GO ontologies “Biological Process,” “Molecular Function,” and “Cellular Component.” Up, upregulated DEGs; DOWN, downregulated DEGs.

For global functional analysis of the DEGs, GO annotation was performed using Blast2GO. Ninety-six of 156 DEGs and 174 of 258 DEGs between ABJ01 and 9# in Daqing and Qiqihar, respectively, were annotated for at least one GO term. The DEGs annotated in GO were grouped into 34 groups based on GO level2 classification (Figures 4A,B). The assigned GO terms belonged to the three main ontologies: ‘biological process’ (BP; 16 and 15 GO terms, respectively), ‘cellular component’ (CC; 10 GO terms), and ‘molecular function’ (MF: 8 and 9 GO terms, respectively). In the BP category, the terms ‘metabolic process’ (51 and 117 DEGs annotated, respectively), ‘cellular process’ (44 and 95 DEGs, respectively), ‘single-organism process’ (36 and 75 DEGs, respectively), and ‘response to stimulus’ (14 and 26 DEGs, respectively) were predominant. In the CC category, ‘cell part’ and ‘cell’ (40 and 100 DEGs, respectively) were the two main terms, followed by ‘organelle’ (31 and 93 DEGs, respectively). In the MF category, the most common terms were ‘catalytic activity’ (56 and DEGs, respectively) and ‘binding’ (46 and 105 DEGs, respectively).

However, the GO functional annotations show that 695 and 742 DEGs out of 1,049 and 1,086 DEGs between Daqing and Qiqihar in ABJ01 and 9# were annotated into 44 and 41 groups based on GO level2 classification, respectively (Figures 4C,D). The encoded proteins belonged to the three main ontologies: ‘BP’ (20 and 18 GO terms, respectively), ‘CC’ (12 and 13 GO terms, respectively), and ‘MF’ (11 GO terms). In BP, ‘metabolic process’ (425 DEGs annotated, both), ‘cellular process’ (344 and 376 DEGs, respectively), ‘single-organism process’ (293 and 314 DEGs, respectively), and ‘response to stimulus’ (137 and 162 DEGs, respectively) were the predominant terms. In the CC category, ‘cell part and cell’ (284 and 296 DEGs, respectively) as the primary term, followed by ‘organelle’ (224 and 223 DEGs, respectively). In the MF category, the most common terms were ‘catalytic activity’ (401 and 392 DEGs, respectively) and ‘binding’ (368 and 380 DEGs, respectively). These results show that the GO functional annotations of genes that were differentially expressed between the transgenic and non-transgenic poplars basically overlapped with those from the different environments. However, there were many more GO terms in the DEGs from the different environments than in the transgenic vs. non-transgenic poplar comparison. There were 14 and 26 DEGs in comprise of DA vs. DB and QA vs. QB from the two environments that were assigned to the ‘response to stimulus’ GO term, and they may be associated with increased stress resistance in the transgenic poplars due to the expression of *JERF36*.

**FIGURE 5:**
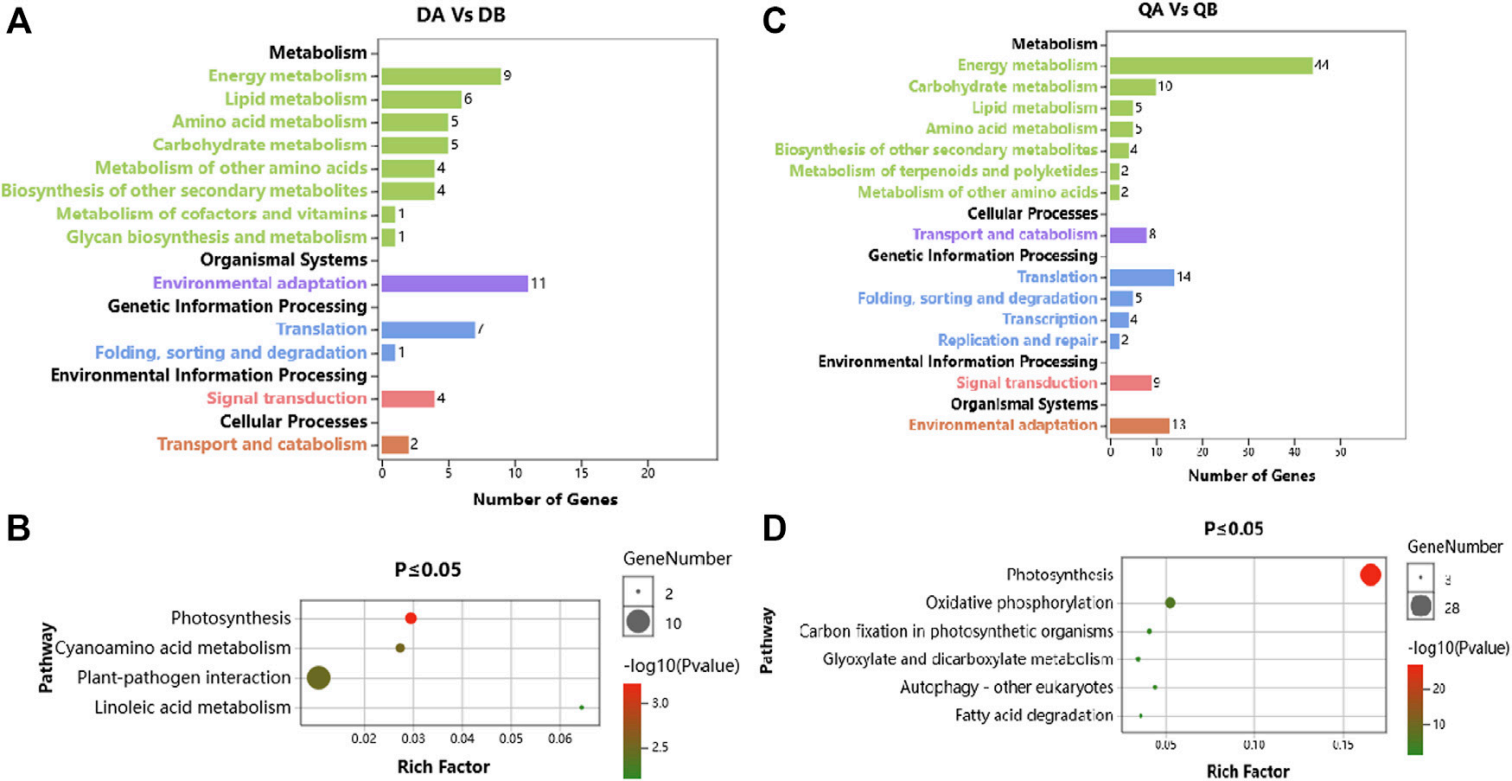
KEGG pathway annotation of DEGs from the comparison of the ABJ01 and 9# transcriptomes. **(A)**. KEGG pathway annotation of ABJ01 vs. 9# in Daqing; **(B)**. Pathway enrichment of ABJ01 vs. 9# in Daqing at *p* ≤ 0.05; **(C)**. KEGG pathway annotation of ABJ01 vs. 9# in Qiqihar; **(D)**. Pathway enrichment of ABJ01 vs. 9# in Qiqihar at *p* ≤ 0.05.

In addition, the biochemical pathways associated with the DEGs were identified by searching the KEGG database. We used a hypergeometric test to identify those pathways that were significantly affected at *p* ≤ 0.05 relative to the whole poplar transcriptome background. There were 46 and 107 DEGs out of 156 and 258 DEGs between ABJ01 and 9# in Daqing and Qiqihar, respectively, that were categorized into 45 and 53 pathways, which were classified into 13 and 14 level B KEGG classification. These DEGs are mainly involved in ‘Energy metabolism’, ‘Environmental adaptation’ and ‘Translation’ (Figures 5A,C). Significant enrichment analysis showed that, in the DA vs. DB comparison, 21 DEGs were enriched in four pathways, including Photosynthesis (5 DEGs), Cyanoamino acid metabolism (4 DEGs), Plant-pathogen interaction (10 DEGs), and Linoleic acid metabolism (2 DEGs) (Figure 5B). Otherwise, in the QA vs. QB comparison, 63 DEGs were enriched in six pathways, which were Photosynthesis (28 DEGs), Oxidative phosphorylation (12 DEGs), and other pathways present in photosynthetic organisms (Figure 5D). These results indicate that the DEGs between the transgenic and non-transgenic poplars involve fewer metabolic pathways, mainly in photosynthesis and plant-fungus interactions, which may be one of the reasons why transgenic poplars grow better than non-transgenic poplars in the different environments.

**FIGURE 6:**
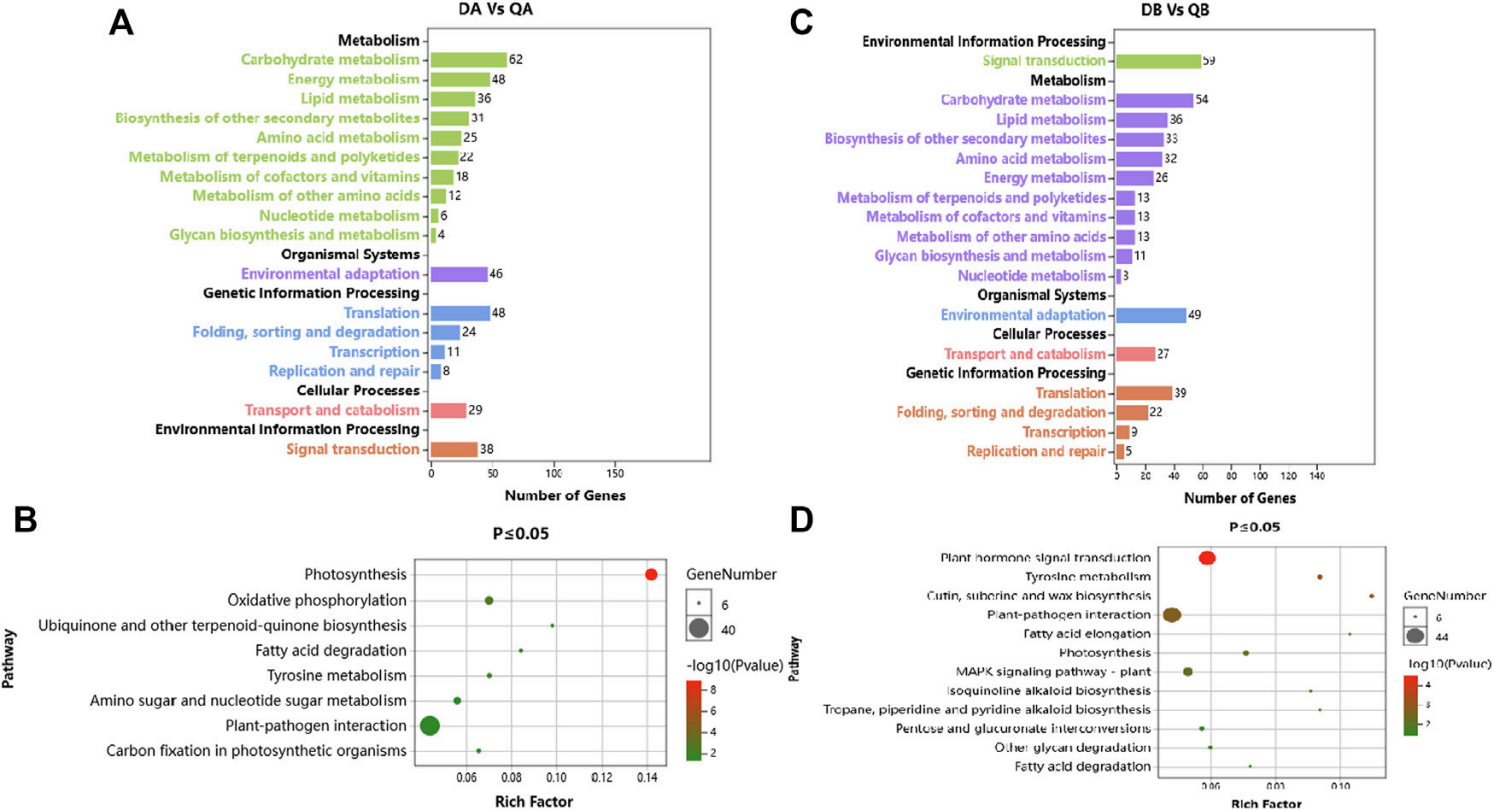
KEGG annotation of DEGs from a comparison of the transcriptomes of proplar trees grown at Daqing with trees from Qiqihar. **(A)**. KEGG pathway annotation of Daqing vs. Qiqihar for ABJ01; **(B)**. Pathway enrichment of Daqing vs. Qiqihar for ABJ01 at *p* ≤ 0.05; **(C)**. KEGG pathway annotation of Daqing vs. Qiqihar for 9#; **(D)**. Pathway enrichment of Daqing vs. Qiqihar for 9# at *p* ≤ 0.05.

In addition, 370 and 345 DEGs out of 1,049 and 1,086 DEGs between Daqing and Qiqihar in ABJ01 and 9# were categorized into 110 and 108 pathways, which classified into 17 level B KEGG annotation (Figures 6A,C). In the DA vs. QA comparison, the DEGs were mainly involved in the KEGG categories ‘Carbohydrate metabolism’, ‘Energy metabolism’, ‘Translation’, and ‘Environmental adaptation’ (Figure 6A). The DEGs showed significant enrichment in pathways such as Photosynthesis (24 DEGs), Plant-pathogen interaction (40 DEGs), Oxidative phosphorylation (16 DEGs), and Amino sugar and nucleotide sugar metabolism (14 DEGs) (Figure 6B). In the DB vs. QB comparison, the DEGs were mainly involved in the KEGG categories ‘Signal transduction’, ‘Carbohydrate metabolism’, ‘Environmental adaptation’, and ‘Energy metabolism’ (Figure 6C). The DEGs showed significant enrichment in pathways such as Plant hormone signal transduction (42 DEGs), Plant-pathogen interaction (44 DEGs), MAPK signaling pathway–plant (23 DEGs), Pentose and glucuronate interconversions (12 DEGs), and Photosynthesis (12 DEGs) (Figure 6D). These results show that the DEGs are involved in many pathways between the different environments in the transgenic and non-transgenic poplars. Moreover, the pathways involved are very similar, and most of them are related to signal transduction and environmental adaptation, which may be clues to the mechanisms by which poplars adapt to environmental changes, with little influence from the introduction of the tomato *JERF36* gene.

### Transcriptional Elements Analysis of Differentially Expressed Genes Between ABJ01 and 9#

In order to evaluate whether DEGs between ABJ01 and 9# were regulated by expression of the tomato *JERF36* transcription factor gene, we searched for the cis-elements that are targets of the *JERF36* transcription factor: DREB (containing the CCGAC core sequence), ABRE (containing the PyACGTGT/GC core sequence), and GCC-box (containing the GCCGCC core sequence) in the promoter regions (2,000 bp upstream of the transcription start sites) of all DEGs between ABJ01 and 9# in Daqing and Qiqihar, using the Phytozome database (http://www.phytozome.net/). The results show that 30 upregulated DEGs (33.33%) and 24 downregulated DEGs (36.36%) from Daqing contain *JERF36* cis-acting elements in their promoter regions. There were 77 upregulated DEGs (42.31%) and 38 downregulated DEGs (50.00%) that contain these cis-acting elements from plants grown in Qiqihar (Table 1). These results show that the upregulated DEGs contain the DREB, ABRE or GCC-box in their promoters at a higher frequency than do the downregulated DEGs. The highest proportion of DEGs were found to contain DREB elements, and the differential expression of these genes may be regulated by the expression of the *JERF36* gene*.*

**TABLE 1:** Types and numbers of cis-elements present in the promoter regions of the DGEs.

Location	Up/Down regulated	Numbers of DGEs						
		Total DEGs	With *cis*-element	GCC-box (%)	ABRE (%)	DREB (%)	With 2 *cis*-elements	With 3 *cis*-elements
Daqing	Up	90	30 (33.33%)	9 (10.00%)	5 (5.56%)	22 (24.44%)	4 (4.44%)	1 (1.11%)
	Down	66	24 (36.36%)	5 (7.57%)	3 (4.55%)	21 (31.82%)	5 (7.57%)	0
Qiqihar	Up	182	77 (42.31%)	10 (5.49%)	15 (8.24%)	67 (36.81%)	13 (7.14%)	1 (0.55%)
	Down	76	38 (50.00%)	9 (11.84%)	8 (10.53%)	25 (32.89%)	4 (5.26%)	0

**FIGURE 7:**
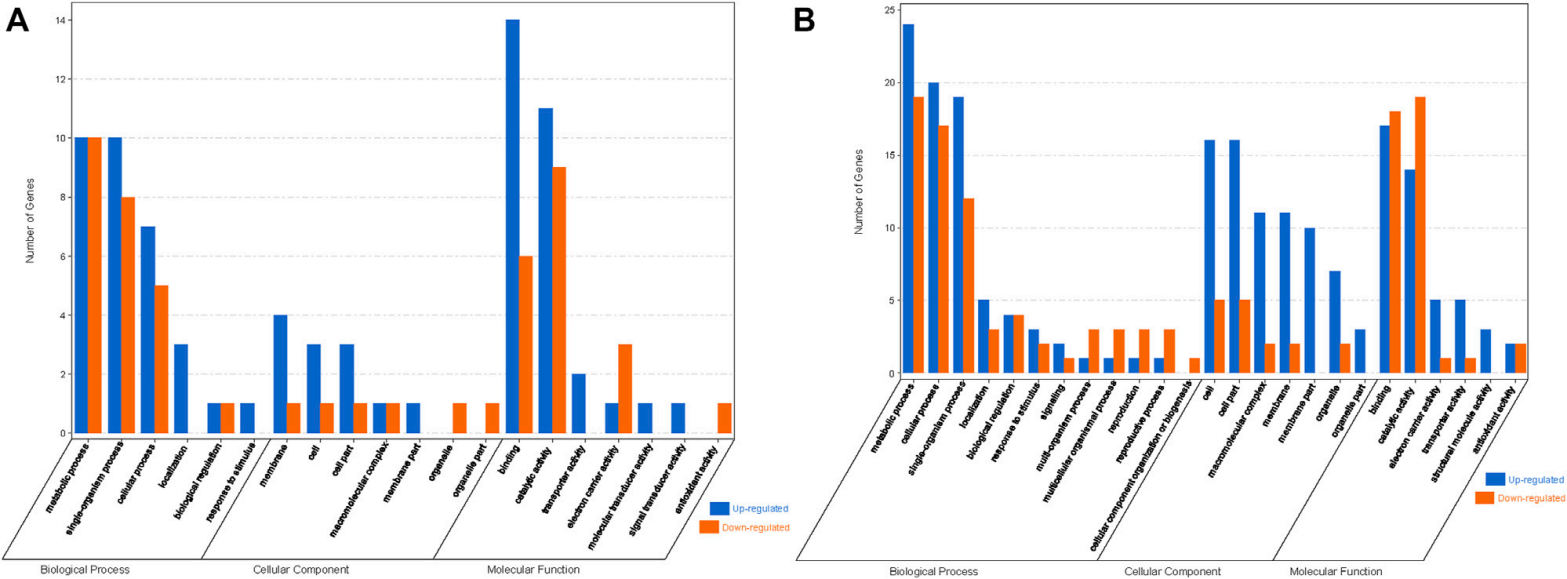
GO enrichment analysis of DEGs with JERF-binding cis-elements in their promoter regions. **(A)**. ABJ01 vs. 9# in Daqing; **(B)**. ABJ01 vs. 9# in Qiqihar. Up, upregulated DEGs; DOWN, downregulated DEGs.

GO enrichment analysis of these DEGs showed that the significantly enriched GO terms mainly included “metabolic process,” “single organism process,” “cell process,” “biofilm,” “cell,” “cell component,” “binding function,” “catalytic activity,” and “transport activity” (Figure 7).

KEGG annotation analysis of these DEGs show that, in Daqing, there were three upregulated genes and three downregulated gene (*Potri.005G009100*, *Potri.013G015700*, *Potri.018G136700*, *Potri.005G041300*, *Potri.005G217000*, *Potri.T032800*) are involved in plant-pathogen interactions, three upregulated and two downregulated genes (*Potri.010G020600*, *Potri.017G103700*, *Potri.T121900*, *Potri.001G388800*, *Potri.011G150300*) participate in Biosynthesis of secondary metabolites, and one upregulated genes and two downregulated genes (*Potri.019G028200*, *Potri.013G143200*, *Potri.013G075000*) that take part in Photosynthetic related metabolic pathways, and (Supplementary Table S3). In the Qiqihar region, There were 14 9 upregulated genes are involved in energy metabolism, among which 9 genes (*Potri.008G207300*, *Potri.011G113700*, *Potri.013G138300*, *Potri.013G141800*, *Potri.013G143200*, *Potri.016G094200*, *Potri.017G052700*, *Potri.019G028100*, *Potri.T005700*) are closely related to Photosystem II, Photosystem I, and ATPase in the photosynthesis pathway: *PsbA*, *PsbC*, *PsbB*, *PsaA*, *PsaB*, *PetA*, and *beta* (Supplementary Table S4, Supplementary Figure S1). Four upregulated genes (*Potri.011G074300*, *Potri.011G074400*, *Potri.012G047500*, *Potri.013G136600*) participate in ribosome pathways. and four genes (one upregulated and three downregulated) take part in signaling pathway such as the MAPK and Plant hormone signal transduction signaling pathway (Supplementary Table S4). Based on the above analyses, we speculated that the differential expression of these genes in photosynthesis, signaling, and plant-pathogen interaction pathways may promote growth and stress tolerance in transgenic poplar.

### Effect of Transgenic Expression of *JERF36* on Gene Expression

**FIGURE 8:**
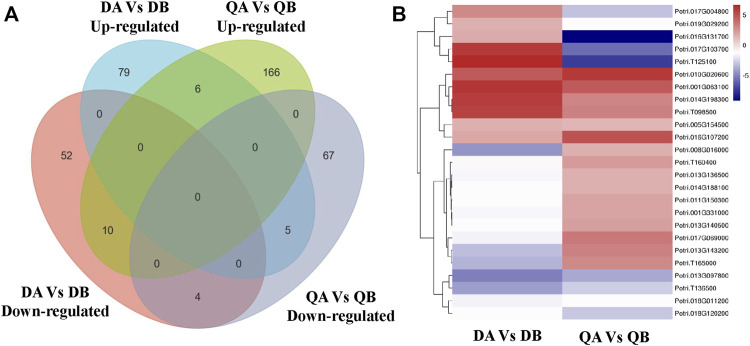
Venn diagram **(A)** and heatmap **(B)** showing co-expressed DEGs in the DA vs. DB and QA vs. QB comparisons.

In order to study the effects of the expression of a foreign gene on changes in transgenic poplar at the transcriptional level, we analyzed the co-expressed DEGs in the DA vs. DB and QA vs. QB comparisons. Ten co-expressed DEGs were found in the two comparisons; among them, 10 DEGs showed the same expression patterns, including six upregulated and four downregulated (Figure 8). Thus, it appears that the differences in expression of these 10 genes are caused by the introduction of the foreign genes *JERF36*. Also, the expression of two upregulated DEGs (*Potri.010G020600* and *Potri.014G198300*) and two downregulated DEGs (*Potri.T136500* and *Potri.018G120200*) that all contain DREB *cis*-elements in their promoter regions was regulated by the expression of *JERF*36 in the transgenic line (Table 2). Functional annotation of these 10 DEGs showed that six upregulated DEGs encode proteins with various predicted functions; translation initiation factor, photosystem II reaction core protein, enzyme activity, nucleotide binding, and monocarboxylic acid metabolism. There were four downregulated DEGs annotated as functioning in phosphate compound metabolism, response to stimulus, and transition metal ion binding. At same time, the DEGs are predicted to play key roles in pathways involving Protein kinases and Photosynthesis proteins. These results suggest that the upregulated or downregulated expression of these genes may explain why transgenic poplars adapt to environment change and grow better than do non-transgenic poplars. For example, expression of *Potri.005G154500* is upregulated in ABJ01, which is annotates to two key proteins of photosystem II, PsbK and PsbI.

**TABLE 2:** Analysis of DGEs induced by the introduction of the *JERF36* gene.

Gene ID	Cis-elements	Log2 fold-change		Funcation annonation	Metaboli pathway
		DA/DB	QA/QB		
Potri.001G063100		6.1116	4.6292	Eukaryotic translation initiation factor 3 subunit 7 family protein, Populus EST from mild drought-stressed leaves	Translation
					RNA transport
					K03251: translation initiation factor 3 subunit D
Potri.014G198300	DREB	5.7801	3.1459	Populus EST from severe drought-stressed opposite wood, regulator of chromosome condensation (rcc1) repeat (rcc1)	Folding, sorting and degradation
					Ubiquitin mediated proteolysis
					K10615: E3 ubiquitin-protein ligase HERC4
Potri.T098500		5.6238	3.1856	Reticulon family protein	Metabolism
					Protein phosphatases and associated proteins
					K18999: RNA polymerase II C-terminal domain phosphatase-like 3/4
Potri.010G020600	DREB	4.6649	5.8961	Shikimate dehydrogenase, oxidoreductase activity	Amino acid metabolism
					Phenylalanine, tyrosine and tryptophan biosynthesis
					K13832: 3-dehydroquinate dehydratase/shikimate dehydrogenase
Potri.016G107200		1.8399	4.9732	Similar to alpha-amylase inhibitor alpha subunit	Signal transduction
					MAPK signaling pathway
					K04730: interleukin-1 receptor-associated kinase 1
Potri.005G154500		1.4352	1.4067	Photosystem ii reaction center protein k	Energy metabolism
					Photosynthesis
					K02712: photosystem II PsbK protein; K02710: photosystem II PsbI protein
Potri.013G097800		−5.1326	−3.8559	Leucine-rich repeat-containing protein	K19613: leucine-rich repeat protein Signal transduction
					Ras signaling pathway
					SHOC2; K03283: heat shock 70 kDa protein 1/8; K06758: L1 cell adhesion molecule like protein
Potri.T136500	DREB	−4.2353	−2.7091	Glycerophosphodiester phosphodiesterase/Glycerophosphoryl diester phosphodiesterase	Signal transduction
					MAPK signaling pathway
					K04733: interleukin-1 receptor-associated kinase 4
Potri.018G011200		−1.6301	−1.3335	Traf-like family protein-related, transition metal ion binding	Metabolism
					Peptidases and inhibitors
					K11855: ubiquitin carboxyl-terminal hydrolase 36/42
Potri.018G120200	DREB	−1.4158	−2.9838	Transmembrane protein ddb, cell-cell junction	-

### Effect of the Environment on Gene Expression

**FIGURE 9:**
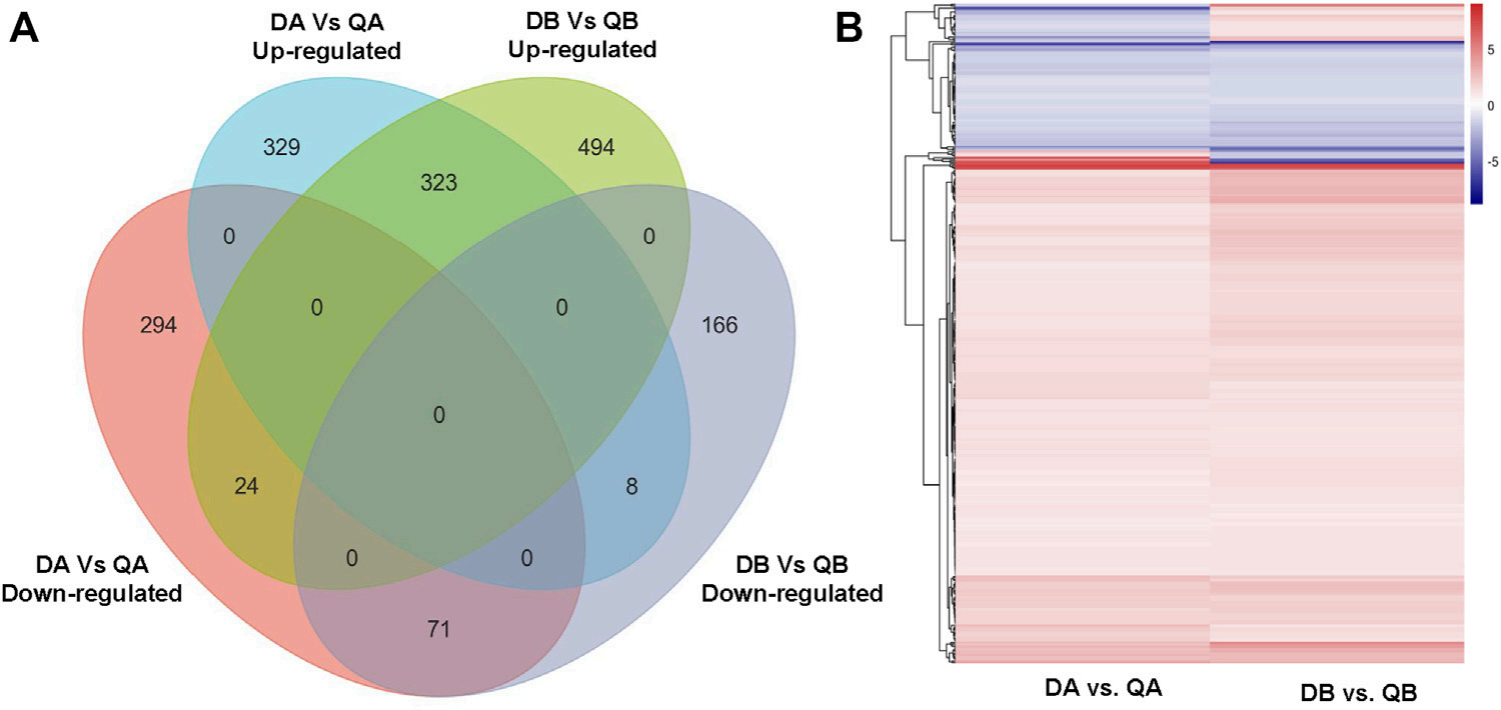
Venn diagram **(A)** and heatmap **(B)** showing co-expressed DEGs in the DA vs. QA and DB vs. QB comparisons.

**FIGURE 10:**
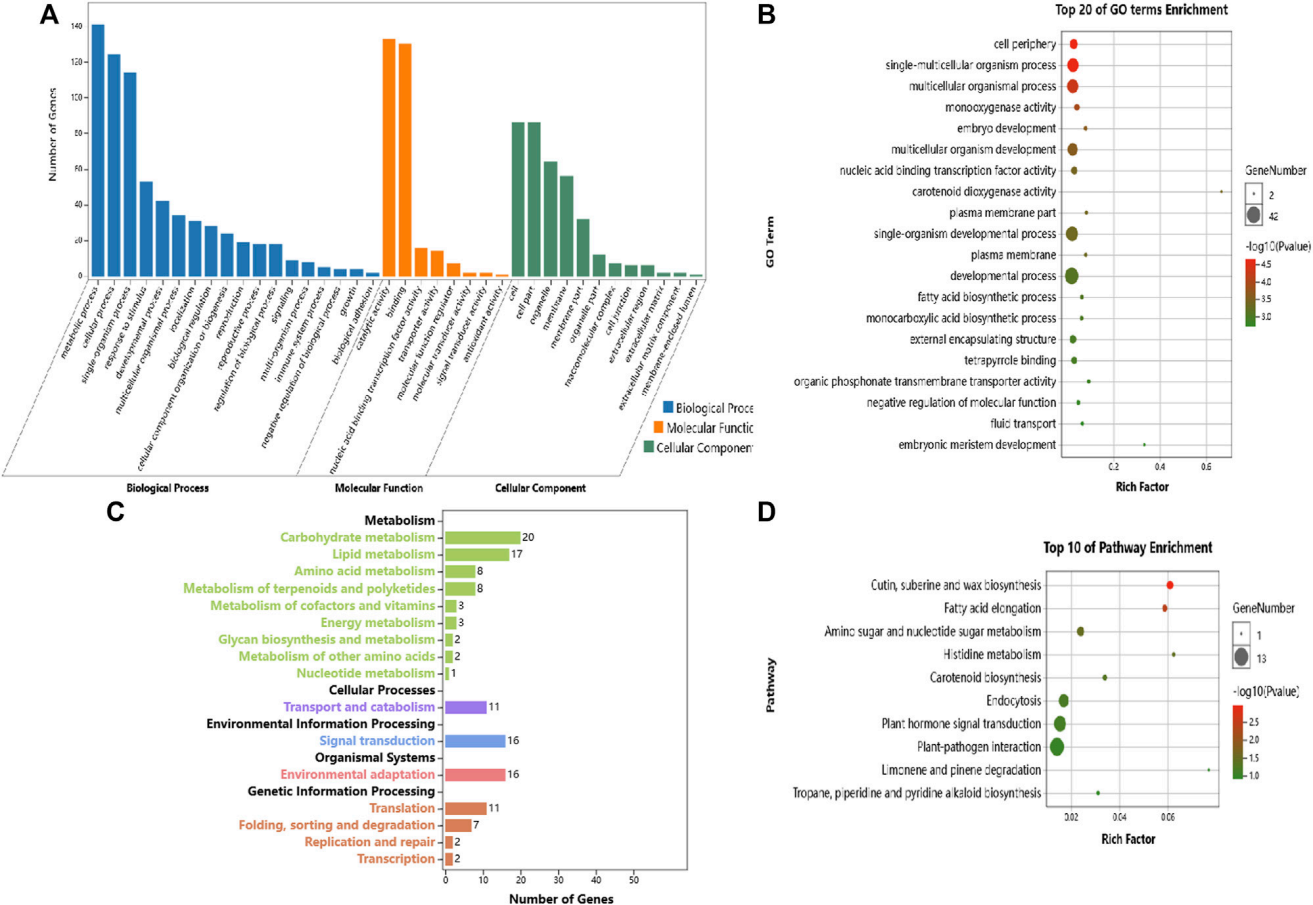
GO and KEGG enrichment analysis of the DEGs that are due to the different environmental conditions at Daqing and Qiqihar. **(A)**. GO annotation analysis of DEGs due to environmental conditions, **(B)**. Top 20 GO Term enrichment of DEGs due to environmental conditions, **(C)**. KEGG enrichment analysis of DEGs due to environmental conditions; **(D)**. Top 10 Pathway enrichment of DEGs due to environmental conditions.

Our objective was to study the genes in poplars that showed differential expression due to environmental differences. Therefore, we identified the DEGs between Daqing and Qiqihar that were shared between ABJ01 and 9#. There were total of 426 differentially expressed genes, of which 323 genes were upregulated in both ABJ01 and 9# in the Daqing vs. Qiqihar comparison, and 71 genes were downregulated (Figure 9), suggesting that a large number of upregulated genes are important for transgenic and non-transgenic poplars to effectively adapt to salt-alkali stress. Our data strongly indicates that the differences in the expression of these 394 genes are caused by the differences in the two environments. GO annotation showed that the DEGs are mainly enriched in developmental processes and signal process, among others (Figures 10A,B). KEGG analysis show that 49 DEGs are mainly annotated in Carbohydrate metabolism, Environmental adaptation, Signal transduction, and Lipid metabolism. For example, 13 were enriched in Plant-pathogen interaction (ko04626), 11 genes were enriched in Plant hormone signal transduction, nine upregulated genes were enriched in Endocytosis, and six were enriched in Amino sugar and nucleotide sugar metabolism (Figures 10C,D). This suggests that the numerous upregulated genes in the transgenic and non-transgenic poplars grown in Daqing may play a key role in the adaptation to salt-alkali stress.

### Interactions Between the Effects of Foreign Gene Introduction and the Environment on Gene Expression

In addition to the DEGs with the same expression pattern mentioned above, we also identified 47 DEGs that showed opposite expression patterns. Firstly, 15 DEGs with opposite expression patterns were shared in the ABJ01 vs. 9# comparisons at Daqing and Qiqihar (Figure 7), and included five genes with upregulated expression in Daqing that were downregulated in Qiqihar, and 10 genes that were downregulated in Daqing but were upregulated in Qiqihar (Table 3). In addition, eight DEGs contained DREB cis elements in their promoter regions. We think that these DEGs between transgenic poplar and non-transgenic poplar are affected by the environmental differences as well as being affected by expression of the introduced *JERF36* gene. Secondly, there were 32 co-expressed DEGs in the comparison of the two fields (Figure 9) that had opposite expression patterns both in ABJ01 and 9#. Among them, eight genes were upregulated in ABJ01 and were downregulated in 9#, and 24 genes that were downregulated in ABJ01 were upregulated in 9# (Table 4). Moreover, 20 DEGs contain ABRE cis elements in their promoter regions. We thought that the expression of the these DEGs between the two environments was not only affected by environmental changes, but also by the expression of the introduced *JERF36* gene. Therefore, there were 47 genes in which expression was affected by the introduced foreign gene and the environment. Annotation of these genes suggests that they mainly encode Ethylene-responsive transcription factors (*Potri.001G203600*, *Potri.013G139200*, and *Potri.T120500*), proteins with photosynthetic functions (*Potri.001G331000* and *Potri.013G143200*), transporter proteins (*Potri.019G029200*, *Potri.T125100*, *Potri.013G139800*, and *Potri.013G139900*), stress or hormone response proteins (*Potri.017G004800*, *Potri.016G131700*, and *Potri.002G211400*), and receptor or hormone signaling pathway proteins (*Potri.017G103700*, *Potri.017G069000*, and *Potri.011G074200*).

**TABLE 3:** Analysis of DGEs induced by the environment in comparisons of the transcriptomes of ABJ01 and 9# from Daqing and Qiqihar.

Gene ID	Cis-elements	Log2 fold-change		Function annotation	Metabolic pathways
		DA/DB	QA/QB		
Potri.T125100	DREB, ABRE	6.6203	−7.3169	Vesicle transport V-snare 12, V-snare 13-like	Folding, sorting and degradation
					SNARE interactions in vesicular transport
					K08493: vesicle transport through interaction with t-snares 1
Potri.017G103700	DREB	5.9852	−5.7767	Acetyl-coa c-acyltransferase/beta-ketothiolase, EST from mild drought-stressed leaves	Lipid metabolism
					Alpha-Linolenic acid metabolism
					K07513: acetyl-coa acyltransferase 1
Potri.017G004800	GCC-box, DREB	2.9326	−3.0486	2-alkenal reductase [nad(p) (+)]/nadph:2-alkenal alpha,beta-hydrogenase	-
Potri.016G131700		1.6705	−9.1405	Protein LURP-one-related 1, Protein LURP-one-related 15	-
Potri.019G029200		1.5601	−1.3831	Populus EST from severe drought-stressed leaves, V-type proton atpase subunit E	Energy metabolism
					Oxidative phosphorylation
					K02150: V-type H+-transporting atpase subunit E
Potri.008G016000		−4.5071	1.4407	Hypothetical protein	
Potri.T165000	DREB	−3.4670	2.8997	Vesca probable cytochrome c	Energy metabolism
					Oxidative phosphorylation
					K08738: cytochrome c
Potri.013G143200	DREB	−3.2091	3.2193	Photosystem II protein D1 (chloroplast), Photosynthetic electron transport chain	Energy metabolism
					Photosynthesis
					K02703: photosystem II P680 reaction center D1 protein
Potri.017G069000	DREB	−1.7145	3.5607	Hydrolase activity, acting on ester/glycosyl bonds	-
Potri.001G331000		−1.5591	1.9762	Photosystem II D2 protein	Energy metabolism
					Photosynthesis
					K02706: photosystem II P680 reaction center D2 protein
Potri.013G136500		−1.4546	1.5280	Ribosomal protein l2	Translation
					Ribosome
					K02886: large subunit ribosomal protein L2
Potri.013G140500		−1.4158	2.0607	50s/60s ribosomal protein l16	Translation
					Ribosome
					K02878: large subunit ribosomal protein L16
Potri.014G188100		−1.2439	1.4739	Beta-fructofuranosidase/saccharase	-
Potri.011G150300	DREB	−1.1683	1.9033	Flavonol synthase/flavanone 3-hydroxylase	Biosynthesis of other secondary metabolites
					Flavonoid biosynthesis
Potri.T160400	DREB	−1.0230	2.3516	Salvia miltiorrhiza mitochondrion	-

**TABLE 4:** Analysis of DGEs induced by introduction of the *JERF36* gene in comparisons of the transcriptomes of Daqing to Qiqihar from ABJ01 and 9#.

Gene ID	Cis-elements	log2 fold-change		Functional annotation	Metabolic pathways
		DA/QA	DB/QB		
Potri.016G131700	ABRE	9.0735	−1.7376	Protein LURP-one-related 1-related	-
Potri.T125100	ABRE	7.1183	−6.8190	Vesicle transport through interaction with t-snares	Folding, sorting and degradation
					SNARE interactions in vesicular transport
					K08493: vesicle transport through interaction with t-SNAREs 1
Potri.017G103700		6.4832	−5.2788	Acetyl-CoA C-acyltransferase/Beta-ketothiolase	Lipid metabolism
					Fatty acid degradation
					K07513: acetyl-CoA acyltransferase 1
Potri.001G203600	ABRE	3.5878	−5.1886	Protein kinase superfamily protein/signal transduction	Signal transduction
					MAPK signaling pathway
					K04733: interleukin-1 receptor-associated kinase 4; K04730:interleukin-1 receptor-associated kinase 1
Potri.017G004800	ABRE	3.1046	−2.8765	2-Alkenal reductase [NAD(P) (+)]/NADPH:2-alkenal Alpha, Beta-hydrogenase	Oxidoreductases
					K07119: uncharacterized protein
Potri.005G103900	ABRE	1.7994	−1.3289	Palmitoyltransferase ZDHHC12-related	Metabolism
					K18932: palmitoyltransferase
Potri.019G029200	ABRE	1.3591	−1.5840	Vacuolar atpase subunit	Energy metabolism
					Oxidative phosphorylation
					K02150: V-type H+-transporting ATPase subunit E
Potri.015G091600		1.0819	−1.9830	AMINO ACID TRANSPORTER	Digestive system
					Protein digestion and absorption
					K14209: solute carrier family 36 (proton-coupled amino acid transporter)
Potri.005G154600		−5.9335	1.8928	F-type H+-transporting ATPase subunit alpha	Energy metabolism
					Photosynthesis/Oxidative phosphorylation
					K02111: F-type H+-transporting ATPase subunit alpha
Potri.T162500		−5.7677	2.1551	Wound-induced protein (DUF3774)	-
Potri.013G143200		−3.8265	2.6019	Photosynthetic reaction centre protein (Photo_RC)	Energy metabolism
					Photosynthesis
					K02703: photosystem II P680 reaction center D1 protein
Potri.017G069000	ABRE	−2.4131	2.8622	Hydrolase activity	-
Potri.005G132300	ABRE	−2.3939	1.2134	Populus tremuloides 26S ribosomal RNA gene	-
Potri.013G139800	ABRE	−2.3780	1.0903	Cell wall-associated hydrolase	
Potri.013G140100	ABRE	−2.3689	1.1475	F-type H+-transporting ATPase subunit alpha	Energy metabolism
					Oxidative phosphorylation
					K02132: F-type H+-transporting ATPase subunit alpha
Potri.013G139200	ABRE	−1.9077	1.0762	Plastid	-
Potri.002G150100	ABRE	−1.8203	2.6872	Geraniol 8-hydroxylase	Metabolism of terpenoids and polyketides
					Diterpenoid biosynthesis
					K16084: ent-cassa-12,15-diene 11-hydroxylase
Potri.013G082900	ABRE	−1.6972	4.8052	Complex subunit 7 homolog-like, transcript variant 3	Translation
					RNA transport
					K13176: THO complex subunit 7
Potri.010G059700	ABRE	−1.5799	2.2994	Acting on a sulfur group of donors, NAD(P) as acceptor/nucleoredoxin 1-RELATED	Metabolism
					Protein phosphatases and associated proteins
					K17609: nucleoredoxin
Potri.013G140500	ABRE	−1.5786	1.8978	Large subunit ribosomal protein L16	Translation
					Ribosome
					K02878: large subunit ribosomal protein L16
Potri.002G211400		−1.5643	1.2444	Oxidoreductase activity	-
Potri.014G188100		−1.4663	1.2514	Beta-fructofuranosidase/Saccharase	-
Potri.001G331000	ABRE	−1.3875	2.1478	Photosystem II P680 reaction center D2 protein	Energy metabolism
					Photosynthesis
					K02706: photosystem II P680 reaction center D2 protein
Potri.011G074200		−1.3468	1.0861	Small subunit ribosomal protein S19	Translation
					Ribosome
					K02965: small subunit ribosomal protein S19
Potri.011G034200	ABRE	−1.3238	1.8344	Interleukin-1 receptor-associated kinase 4	Signal transduction
					MAPK signaling pathway
					K04733: interleukin-1 receptor-associated kinase 4
Potri.T069900		−1.1957	5.9234	Potassium channel tetramerisation domain containing protein	Genetic information processing
					K15074: BTB/POZ domain-containing adapter for CUL3-mediated RhoA degradation protein
Potri.013G142200		−1.1540	1.2669	DNA-directed RNA polymerase subunit beta	Transcription
					RNA polymerase
					K03043: DNA-directed RNA polymerase subunit beta
Potri.T120500	ABRE	−1.1362	1.3053	Universal stress protein family (Usp)	-
Potri.002G040000		−1.1221	1.0489	Photosystem I P700 chlorophyll a apoprotein A1	Energy metabolism
					Photosynthesis
					K02689: photosystem I P700 chlorophyll a apoprotein A1
Potri.013G139900	ABRE	−1.0229	1.1832	Retrotransposon protein	-
Potri.013G143100		−1.0162	1.3197	-	
Potri.013G141800	ABRE	−1.0128	1.3093	Photosystem I P700 chlorophyll a apoprotein A1	Energy metabolism
					Photosynthesis
					K02689: photosystem I P700 chlorophyll a apoprotein A1

### Validation of RNA Sequencing Results by qRT-RCR

**FIGURE 11:**
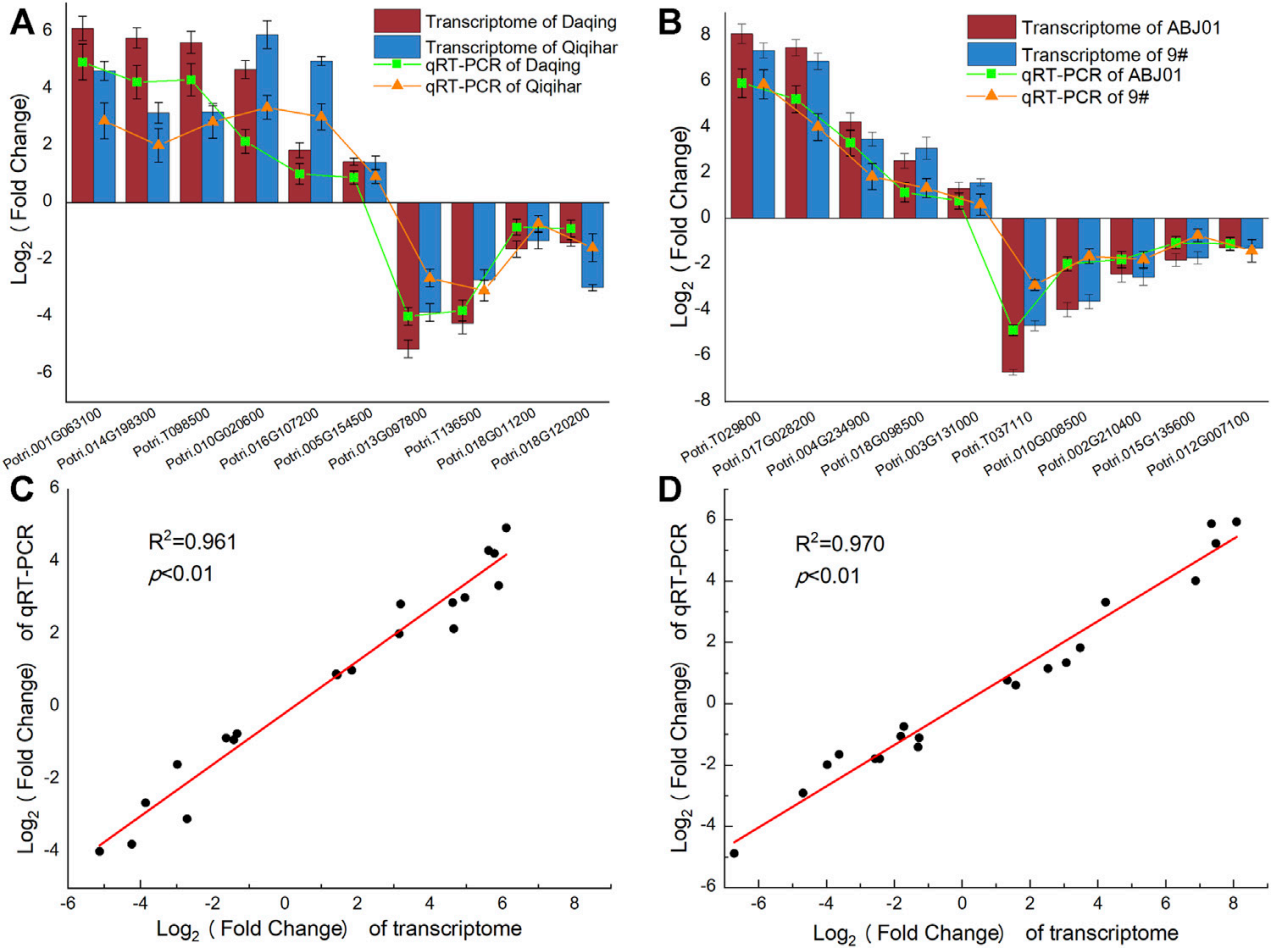
Expression pattern of 20 DEGs by qRT-PCR and transcriptome sequencing. **(A)**. Log_2_ Fold change (ABJ01/9#) in Daqing and Qiqihar; **(B)**. Log_2_ Fold change (Daqing/Qiqihar) in ABJ01 and 9#; **(C)** correlation o Log_2_f fold change (ABJ01/9#) in Daqing and Qiqihar analyzed by RNA-seq (x-axis) with data obtained using qRT-PCR (y-axis); **(D)** correlation of Log_2_ fold change (Daqing/Qiqihar) in ABJ01 and 9# analyzed by RNA-seq (x-axis) with data obtained using qRT-PCR (y-axis).

To determine the reliability of the expression of DEGs obtained from Illumina RNA-seq data, 10 genes (six upregulated and four downregulated genes) identified in the DA vs. DB and QA vs. QB comparisons and 10 genes (five upregulated and five downregulated genes) identified in the DA vs. QA and DB vs. QB comparisons were randomly selected for qRT-PCR analysis. To compare the relative expression, log2 fold-changes were determined between the RNA-seq and qRT-PCR data. As shown in Figure 11, the fold-changes and expression patterns for the 20 DEGs calculated from qRT-PCR data were consistent with the transcriptome sequencing results, with a significant positive correlation (*R* ^2^ > 0.96, *p* < 0.01), indicating that the expression results for the DEGs calculated from the transcriptome sequencing data were reliable.

## Discussion

The recent rapid advances in high-throughput sequencing technology and bioinformatics has resulted in a sharp increase in the number of studies on genome-wide transcriptional changes in transgenic plants, which provide new ideas for studying gene expression and the unexpected effects on transgenic plants (Kuiper et al., 2001; Ouakfaoui and Miki, 2005; Ricroch et al., 2011; Gong and Wang, 2013; Herman and Price, 2013; Pauwels et al., 2015; Wang et al., 2018; Tan et al., 2019; Liu et al., 2020). However, most of this research is focused on crops, and there are few studies on transgenic trees, especially mature transgenic trees. In this study, high-throughput RNA-seq technology was used to transcriptome sequencing and data analysis on mature transgenic poplar trees and non-transgenic receptors under in saline (Daqing) and non-saline (Qiqihar) sites.

Analysis of the DEGs at the transcriptome level showed that, compared to non-transgenic poplar (9#), there were fewer differentially expressed genes in transgenic poplar (ABJ01) both at Daqing and Qiqihar, accounting for 0.77% and 1.31% of the total expressed genes, respectively. Among these DEGs, most of the genes were upregulated, and the expression patterns was basically the same. However, there were about 1,000 DEGs (accounting for 5% of the total expressed genes) identified in the Daqing vs. Qiqihar comparison in both ABJ01 and 9#, and the number of DEGs in the transgenic and non-transgenic poplars from the different sites was very similar. The difference between the transgenic and non-transgenic controls was much smaller than that between the two poplar lines from the different environments. Our results were similar to those from transcriptomic studies in rice, maize, wheat, soybean, and potato. (Catchpole et al., 2005; Batista et al., 2008; Baudo et al., 2006; Cheng et al., 2008; Barros et al., 2010; Kogel et al., 2010; Batista et al., 2017; Fu et al., 2019).

GO and KEGG pathway analyses of the DEGs revealed that, compared to non-transgenic 9#, the GO and KEGG pathway assignments of the DEGs in ABJ01 were similar between Daqing and Qiqihar. However, there were slight differences in the significantly enriched pathways between Daqing and Qiqihar. At Daqing, the DEGs from the ABJ01 vs. 9# comparison mainly showed significant enrichment in Plant-pathogen interaction (seven upregulated and three downregulated DEGs) and Photosynthesis (two upregulated and three downregulated DEGs). Whereas at Qiqihar, the DEGs from the ABJ01 vs. 9# comparison was mainly significantly enriched in Photosynthesis (28 DEGs), Oxidative phosphorylation (12 DEGs), and other pathways present in photosynthetic organisms. Studies have shown that overexpression of many ERF transcription factors, such as those found in poplar, soybean, tomato, wheat, and tobacco, result in stronger tolerance to abiotic and biotic stress by regulating the expression of stress-related genes (Yao et al., 2016; Ku et al., 2018; Ding et al., 2020). This suggests that, compared with non-transgenic poplars, transgenic poplars mainly adapt to salt-alkali stress by regulating the expression of genes related to plant-pathogen interactions and photosynthesis. However, under non-salt stress, transgenic poplars improve their environmental adaptability mainly by improving energy metabolism such as photosynthesis to maintain a higher growth level. Our results showed that the ability of transgenic and non-transgenic poplars to respond to the environment was differed between the two different environments. Transgenic poplar trees (ABJ01) expression the tomato *JERF36* gene show improved tolerance to biotic and abiotic stresses.

Functional analysis of the DEGs between ABJ01 and 9# from Daqing show that, compared to Qiqihar, the GO classifications and Pathway enrichments of the DEGs in the transgenic and non-transgenic poplars grown in saline-alkali soils were quite similar. In addition, more genes were significantly enriched in the Plant-pathogen interaction and photosynthesis pathways. A previous study showed that salt stress leads to various physiological and molecular changes (Van-Zelm et al., 2020). Salt stress affects light-harvesting complex formation and regulates the state transition of photosynthesis (Chen and Hoehenwarter, 2015). This indicates that both transgenic and non-transgenic poplars can better adapt to environmental changes by enhancing the regulation of plant-pathogen interaction and photosynthetic pathways in response to salt-alkali stress. Gene expression pattern analysis showed that gene expression in both ABJ01 and 9# in the plant-pathogen interaction pathway was mainly upregulated. However, the expression pattern of genes in the Photosynthesis pathway was different; gene expression in ABJ01 was mainly downregulated, while it was upregulated in non-transgenic 9#. We speculate that the introduction of *JERF36* gene may affect the regulation of photosynthesis pathway.

In addition, there were more significantly enriched DEGs in Plant hormone signal transduction (42 DEGs), MAPK signaling pathways (23 DEGs), and Pentose and glucuronate interconversions (12 DEGs) in 9#, and their expression was mainly upregulated. Studies have shown that plant hormones regulate plant growth and development, as well as responses to abiotic and biotic stresses (Verma et al., 2016; Yu et al., 2020). MAPKs can be activated by various biotic and abiotic stresses, which is a common in stress response of plants (Zhang et al., 2022). In response to salt stress, signaling molecules such as phosphatidic acid and ROS are activated by MAPKs through nicotinamide adenine dinucleotide phosphate (NADPH)-oxidase, resulting in increased osmolyte synthesis and the accumulation of osmotic compounds (Moustafa et al., 2014; Jalmi and Sinha, 2015). This may be the reason for the increase in sugar content in the salt stress treatment. Of the proteins involved in pentose and glucuronate interconversions, such as pectinesterase, pectate lyase, and polygalacturonase-2, pectate lyase and pectinesterase are involved in the response to osmotic stress (Tang et al., 2020). This indicates that, in order to better respond to salt-alkali stress, more stress-response pathways with up or downregulation of genes may need to be identified in non-transgenic poplars.

Numerous studies have shown that the AP2/ERF family of transcription factors, which are found mainly in plants, play very important roles in regulating diverse environmental stress responses, such as abiotic stresses (cold, heat, drought, salinity, and osmotic stress) and biotic stresses (herbivorous insects and microbial pathogens) (Feng et al., 2020). AP2/ERF transcription factors, such as *JERF36* and *ERF76* have the ability to enhance salt tolerance in transgenic poplar by increasing ABA and GA biosynthesis (Li et al., 2009; Yao et al., 2016; Ding et al., 2020). DREB transcription factor, for example, SALTRESPONSIVE ERF1 (SERF1), can amplify and transmit salt-inducing signals through the MAPK cascade signaling pathway, leading to a response to salt stress in the plant (Schmidt et al., 2013). In our study, analysis of cis-acting elements in the DEGs in transgenic and non-transgenic poplars showed that 30%–50% of them contained cis-elements for *JERF36*, mainly DREB cis-elements in their promoter regions. Functional annotation found that in saline-alkali areas, the DEGs containing cis-elements are mainly involved in photosynthesis and plant pathogen interaction *via* up or downregulation. In non-saline regions, the DEGs containing cis-elements are mainly involved in photosynthesis and MAPK signal transduction. This suggests that transgenic poplars may have enhanced salt tolerance and improved growth due to the number of DEGs in those pathways. However, the different expression patterns in trees from Daqing and Qiqihar may be caused by different mechanisms of ERF regulation in the above metabolic pathways that result from differences between the two environments.

In order to study the effects of foreign gene introduction or environmental differences on the changes in gene expression in transgenic poplar. we identified 10 DEGs (six upregulated and four downregulated) that expressed in both the DA vs. DB and QA vs. QB comparisons, which were due only to the introduction of the foreign gene. We also identified 394 DEGs (323 upregulated and 71 downregulated) that resulted from the environmental difference, and were expressed in both of the DA vs. DB and QA vs. QB comparisons. In addition, 47 DEGs with opposite expression patterns were identified in both the DA vs. DB and QA vs. QB comparisons (15 DEGs) and the DA vs. DB and QA vs. QB comparisons (32 DEGs), which were affected by the interaction between the introduced gene and the environment. This further indicated that the environmental differences had a greater influence on the transcriptome in the transgenic poplars than did the transgenic event. Annotation of 57 DEGs affected by foreign gene introduction (Table 3) or interaction between the introduced gene and the environment (Table 4) showed that the DEGs mainly play central roles in the Photosynthesis and Oxidative phosphorylation (Energy metabolism), MAPK and Plant hormone signaling (Signal transduction), and Ribosome (Translation) pathways. It is well known that, the AP2/ERF super-family of transcription factors also play important roles in hormonal regulation and plant development. Many studies have shown that AP2/ERF-type transcription factors can induce phytohormone responses, such as ethylene, ABA, and jasmonic acid, by activating target genes, other response factors, and even other AP2/ERF transcription factors to regulate various growth processes in plants (Ding et al., 2020; Feng et al., 2020). In plants, MAPKs are activated mainly by stress-triggered secondary signals; for example, ABA and Ca^+^, rather than by the primary osmotic stress signal (Zhu, 2016; Zhao et al., 2021). AP2/ERF transcription factors can participate in lipid synthesis by regulating genes in the fatty acid biosynthesis pathway (Jiang et al., 2018). In the present study, we speculated that the transgenic poplar trees expressing the foreign *JERF36* gene may show enhanced photosynthesis, peroxisome activity, and stress signal transduction by regulating the expression of genes in these pathways, resulting in improved adaptability to salt-alkali stress.

However, stress also induce organellar responses from the chloroplast, mitochondrion, peroxisome, nucleus, and cell wall, as well as signal transduction; examples are ionic stress signaling, osmotic stress signaling (such as lipid signals including phosphatidic acid and phosphoinositides), ABA signaling, cold and heat stress signaling, systemic signaling (such as in plant-pathogen interactions) (Hou et al., 2016; Zhu, 2016), transcriptional regulation, transcript processing, and translational regulation, (Zhang et al., 2022). Therefore, there are some DEGs with different expression patterns in the DA vs. DB and QA vs. QB or DA vs. QA and DB vs. QB comparisons, possibly due to properties of the different environments in Daqing and Qiqihar, such as soil salinity.

Annotation of the 394 DEGs that resulted from differences in the two environments showed that the DEGs mainly participate in carbohydrate metabolism, environmental adaptation, signal transduction, and lipid metabolism. For example, 13 upregulated genes were enriched in plant-pathogen interaction, 11 upregulated genes were enriched in plant hormone signal transduction, and nine upregulated genes were enriched in endocytosis. Studies have shown that sophisticated crosstalk occurs among the different hormones in plant growth adaptation to salt stress, the cooperation or antagonism among the different plant hormones is dependent on growth stages, and plants adapt to salt stress through flexible regulation of hormone levels and/or signaling (Yu et al., 2020). In plants, endocytosis and active endosomal trafficking is essential to maintain cell homeostasis during salt stress (Sanderfoot et al., 2000; Valencia et al., 2016; Wang et al., 2020). Plants can defense against pathogens attacking by oxidative burst production of ROS, the activation of ion fluxes, and MAPK signaling cascades (Jones and Dangi, 2006; Li et al., 2020). In addition, plants are constantly challenged by a combination of abiotic and biotic stresses in the natural environment. Plant hormone signaling such as through Ca^+^ sensors, the ABA-mediated stress response including ABA and JA, ethylene, and SA, as well as phospholipid biosynthesis pathways, always show crosstalk between the biotic and abiotic stress responses (Ku et al., 2018). Above all, in our study, upregulated expression of genes in the stress response pathways, such as plant hormone signal transduction, may enhance the tolerance to salt-alkali stress in both transgenic and non-transgenic poplars. At the same time, the crosstalk between biotic and abiotic stress responses by plant hormones may improve the ability of transgenic and non-transgenic poplars to defend against pathogens.

## Conclusion

The number of genes that showed differential expression due to environmental factors was significantly greater than the number of DEGs that resulted from the introduction of the *JERF36* gene, and the synergistic effect of the environment and the foreign gene was significantly greater than that caused by transgenesis and *JERF36* introduction. Between 30% and 50% of the DEGs in the comparisons of transgenic and non-transgenic poplars contained cis-elements that bind *JERF36*. The different expression patterns in Daqing and Qiqihar may be caused by different mechanisms of ERF regulation in photosynthesis and the MAPK signal transduction pathways due to environmental differences. It is indicated that, the introduction of JERF36 have the potential to improve the salt tolerance of transgenic poplar. We identified 10 DEGs that were due to the effects of the foreign gene introduction, 394 DEGs that resulted from the environmental differences, and 47 DEGs that resulted from the combined effects of the foreign gene introduction and the environmental differences. Transgenic poplar trees expressing the *JERF36* gene may show improvements in photosynthesis, peroxisome activity, and stress signal transduction by regulating the expression of genes in the photosynthesis, oxidative phosphorylation, MAPK and plant hormone signaling, and ribosome pathways, thus enhancing their adaptability to salt-alkali stress. The crosstalk between biotic and abiotic stress responses by plant hormone signaling pathways may improve the defense of transgenic and non-transgenic poplars against pathogens. There were no unexpected effects resulting from the introduction of the *JERF36* gene from tomato on the transgenic poplar trees.

## Data Availability

The original contributions presented in the study are included in the article/Supplementary Material, further inquiries can be directed to the corresponding authors.
