# Supplementary material for: Transcriptomic Analysis of Mature Transgenic Poplar Expressing the Transcription Factor JERF36 Gene in Two Different Environments
Source: Front Bioeng Biotechnol. 2022 Jun 14;10:929681. doi: 10.3389/fbioe.2022.929681 (PMC9237257; doi:10.3389/fbioe.2022.929681)
Supplement: Supplementary file 2 [file Table1.DOCX]

**Table S1 Nucleotide sequences of the primers used for qRT-PCR in this study**

| **Gene ID** | **Forward primer** | **Reverse primer** |
| --- | --- | --- |
| *UBQ-Like* | TGAGGCTTAGGGGAGGAACT | TGTAGTCGCGAGCTGTCTTG |
| Potri.001G063100 | ACTTCACTGCCGATGATTCC | TACGTGCCCTCTCTTTCTCC |
| Potri.005G154500 | ATGATCCAGGGCGTAATCCT | GGTTCCGTTGATGACTTGGT |
| Potri.010G020600 | TGCCGGTGCTGATATTGTTA | CCCTCTCACCCATGACAAGT |
| Potri.014G198300 | TGTTGAGGCATCTCCACTTG | CCAAACTATCGGCATGTCCT |
| Potri.016G107200 | GCCTGCTTAACGGTGCTTAC | CGCGAGTGAGTTGAAGAGAA |
| Potri.T098500 | CTTGGGGAGCTTGTTCTCAC | CCCCTTCTACCTGTGTTGGA |
| Potri.T136500 | TCCGCTCTTAGTTGGGAGAA | GAGCATAGTTTCGCCAGACC |
| Potri.013G097800 | ATCGAACCTGCTCTTTGGAA | CCCCATCTCTTTCATGCACT |
| Potri.018G011200 | TAGCATCGCACTTGTTCCTG | GGGAATCTGGGTTTGTCTGA |
| Potri.018G120200 | CCTTGGCTGGGTTCTTGTTA | TTTCTTGAGCAGTTGCGTTG |
| Potri.T029800 | ATTGCGACTTTGAAGGCTGT | AGCTGGAGTGCGGTAGGTTA |
| Potri.017G028200 | CGCTGCCTTTTTATTTGCTC | CACCTTCTTGGCTCCATCAT |
| Potri.004G234900 | ATGGCTCCACCAATAGCATC | TGGGCAATCCACACAATATG |
| Potri.018G098500 | GCCCCTTTATTTCTCCAACC | GGACTAACCCTGGTGCTCAA |
| Potri.003G131000 | TCTGAATCCGCCTGCTACTT | TCGGGTTGTGGACTATGGTT |
| Potri.T037100 | TGGTAAGGCATTCTCCTTGG | CGGTGTCGGATTCTTTTCAT |
| Potri.002G210400 | TATCCGCAGGAAGCCTAAGA | AGATTGATGTCCCCATCCAC |
| Potri.015G135600 | TACCAAACCAGACCGACCTC | AGCAAGAGACCCACCAGCTA |
| Potri.010G008500 | TGAGGGAGTTGATGATGCAG | TTGGTAATGCACCCTTGTGA |
| Potri.012G007100 | GGCAAACGAAAATTCCTGAG | GATGATAGCAGTGGCAAGCA |
