# Supplementary material for: Transcriptomic Analysis of Mature Transgenic Poplar Expressing the Transcription Factor JERF36 Gene in Two Different Environments
Source: Front Bioeng Biotechnol. 2022 Jun 14;10:929681. doi: 10.3389/fbioe.2022.929681 (PMC9237257; doi:10.3389/fbioe.2022.929681)
Supplement: Supplementary file 3 [file Table2.docx]

**Table S2 RNA-sequencing and transcriptome mapping results in transgenic and non-transgenic poplars**

| Sample | Raw Data Size (bp) | Raw Reads Number | Clean Data Size (bp) | Clean Reads Number | Q20 (%) | Clean Data Rate (%) | Total Mapped Reads (%) | Unique Match Reads (%) | Multi-position Match Reads (%) |
| --- | --- | --- | --- | --- | --- | --- | --- | --- | --- |
| DA-1 | 1206848250 | 24136965 | 1206416800 | 24128336 | 93.4 | 99.96 | 73.73 | 41.62 | 32.11 |
| DA-2 | 1206795350 | 24135907 | 1206485250 | 24129705 | 93.3 | 99.97 | 74.33 | 41.79 | 32.54 |
| DA-3 | 1206858600 | 24137172 | 1205995950 | 24119919 | 92.8 | 99.92 | 73.06 | 40.69 | 32.37 |
| DB-1 | 1206854350 | 24137087 | 1206412500 | 24128250 | 93.3 | 99.96 | 73.46 | 41.46 | 32.00 |
| DB-2 | 1206854000 | 24137080 | 1206479650 | 24129593 | 92.5 | 99.96 | 73.53 | 40.89 | 32.64 |
| DB-3 | 1206856400 | 24137128 | 1206443300 | 24128866 | 92.9 | 99.96 | 69.54 | 38.83 | 30.71 |
| QA-1 | 1206884250 | 24137685 | 1206765550 | 24135311 | 95.7 | 99.99 | 68.62 | 40.01 | 28.61 |
| QA-2 | 1206878150 | 24137563 | 1206752250 | 24135045 | 95.7 | 99.98 | 70.11 | 40.88 | 29.23 |
| QA-3 | 1206884750 | 24137695 | 1206726650 | 24134533 | 95.1 | 99.98 | 74.01 | 42.90 | 31.11 |
| QB-1 | 1206845600 | 24136912 | 1206423800 | 24128476 | 92.7 | 99.96 | 72.04 | 40.03 | 32.01 |
| QB-2 | 1206862400 | 24137248 | 1206295550 | 24125911 | 92.9 | 99.95 | 72.96 | 40.82 | 32.14 |
| QB-3 | 1206846900 | 24136938 | 1206456600 | 24129132 | 93.2 | 99.96 | 72.85 | 40.66 | 32.19 |

DA: ABJ01 from Daqing, DB: 9# from Daqing; QA: ABJ01 from Qiqihar, QB: 9# from Qiqihar.
