# Supplementary figures and images for: Transcriptomic Analysis of Mature Transgenic Poplar Expressing the Transcription Factor JERF36 Gene in Two Different Environments
Source: Front Bioeng Biotechnol. 2022 Jun 14;10:929681. doi: 10.3389/fbioe.2022.929681 (PMC9237257; doi:10.3389/fbioe.2022.929681)

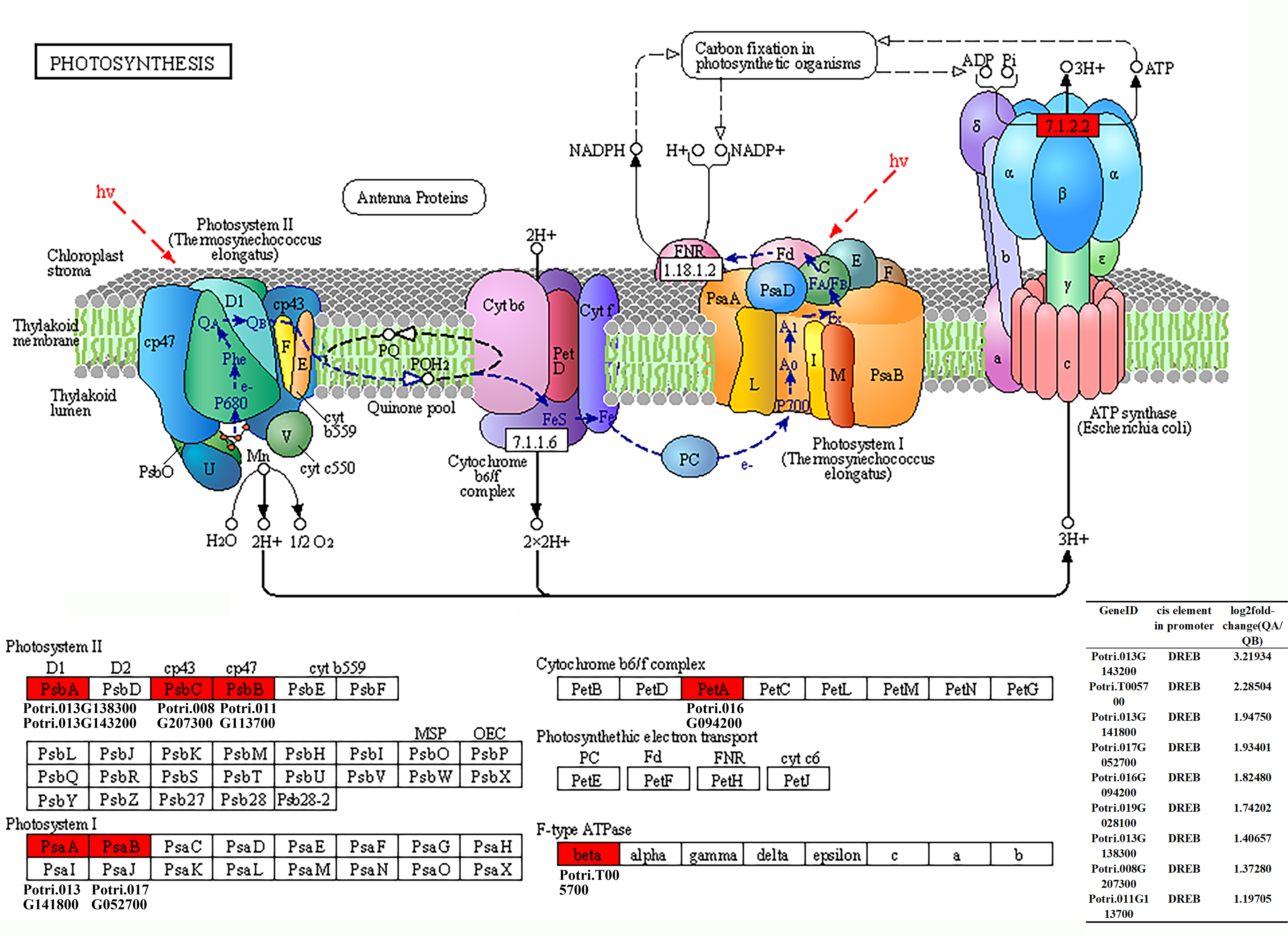

Supplement: Supplementary file 5 [file Image1.PNG]
